# FlexBRDF: A Flexible BRDF Correction for Grouped Processing of Airborne Imaging Spectroscopy Flightlines

**DOI:** 10.1029/2021JG006622

**Published:** 2022-01-24

**Authors:** Natalie Queally, Zhiwei Ye, Ting Zheng, Adam Chlus, Fabian Schneider, Ryan P. Pavlick, Philip A. Townsend

**Affiliations:** ^1^ Department of Forest and Wildlife Ecology University of Wisconsin‐Madison Madison WI USA; ^2^ Jet Propulsion Laboratory California Institute of Technology Pasadena CA USA

**Keywords:** bidirectional reflectance distribution function, BRDF, imaging spectroscopy, AVIRIS, NEON, Ross‐Li kernels

## Abstract

Bidirectional reflectance distribution function (BRDF) effects are a persistent issue for the analysis of vegetation in airborne imaging spectroscopy data, especially when mosaicking results from adjacent flightlines. With the advent of large airborne imaging efforts from NASA and the U.S. National Ecological Observatory Network (NEON), there is increasing need for methods that are flexible and automatable across images with diverse land cover. Flexible bidirectional reflectance distribution function (FlexBRDF) is built upon the widely used kernel method, with additional features including stratified random sampling across flightline groups, dynamic land cover stratification by normalized difference vegetation index (NDVI), interpolation of correction coefficients across NDVI bins, and the use of a reference solar zenith angle. We demonstrate FlexBRDF using nine long (150–400 km) airborne visible/infrared imaging spectrometer (AVIRIS)‐Classic flightlines collected on 22 May 2013 over Southern California, where diverse land cover and a wide range of solar illumination yield significant BRDF effects. We further test the approach on additional AVIRIS‐Classic data from California, AVIRIS‐Next Generation data from the Arctic and India, and NEON imagery from Wisconsin. Comparison of overlapping areas of flightlines show that models built from multiple flightlines performed better than those built for single images (root mean square error improved up to 2.3% and mean absolute deviation 2.5%). Standardization to a common solar zenith angle among a flightline group improved performance, and interpolation across bins minimized between‐bin boundaries. While BRDF corrections for individual sites suffice for local studies, FlexBRDF is an open source option that is compatible with bulk processing of large airborne data sets covering diverse land cover needed for calibration/validation of forthcoming spaceborne imaging spectroscopy missions.

## Key Points

- A flexible bidirectional reflectance distribution function (FlexBRDF) corrects BRDF effects in groups of adjacent flightlines to minimize between‐image spectral differences
- Interpolation of normalized difference vegetation index bins prevents between‐bin edges
- FlexBRDF is highly customizable and performed well for a variety of sites and sensors

## 1 Introduction

Imaging spectroscopy, also known as hyperspectral remote sensing, has become a critical technology for understanding the dynamics of vegetation, including functional traits (Asner, Martin, et al., 2015; Singh et al., 2015; Wang, Chlus, et al., 2020; Wang, Townsend, et al., 2019), vegetation type, composition, and diversity (Colgan et al., 2012; Li et al., 2005; Roberts, Gardner, et al., 1998; Schneider et al., 2017), senescent vegetation fraction (Dennison et al., 2019; Guerschman et al., 2009; Roberts, Smith, et al., 1993), photosynthetic capacity (Serbin et al., 2015), and disturbance and stress (Asner, Brodrick, et al., 2016; Veraverbeke et al., 2014). Current and forthcoming spaceborne missions such as Surface Biology and Geology (SBG; NASEM, 2018), Copernicus Hyperspectral Imaging Mission for the Environment (Nieke & Rast, 2018), Hyperspectral Imager Suite (Iwasaki et al., 2011), PRISMA (Loizzo et al., 2018), and EnMap (Stuffler et al., 2007) will offer unprecedented capacity to characterize Earth's terrestrial ecosystems, which is critical given rapid ongoing losses in biodiversity and climate change (Jetz et al., 2016; Schimel et al., 2020). Calibration and validation of global missions such as SBG will require extensive airborne imaging spectroscopy to provide sufficient spatial and temporal coverage of necessary biological and physical measurements that are not logistically feasible from in‐situ sampling. Such airborne campaigns have increased significantly in recent years, with efforts including the U.S. National Ecological Observatory Network (NEON; Kampe, 2010), and National Aeronautics and Space Administration (NASA) airborne imaging spectroscopy campaigns such as the Arctic boreal vulnerability experiment (Kasischke et al., 2010), India (Bhattacharya et al., 2019) and Europe (Hueni et al., 2018) campaigns, and the California western biodiversity time series (Lee et al., 2015). In addition to providing critical baseline data for satellite missions, airborne missions currently also provide important high resolution data to prototype development of algorithms and data sets for those missions.

The airborne data sets needed to support satellite campaigns have a number of important conditions. First, these airborne campaigns must cover areas larger than a single airborne flightline, necessitating datasets comprised of multiple flightlines covering a flight box over areas of interest. As well, consistent retrieval of spectral surface reflectance is necessary for producing reliable and comparable maps of derived data products, both across multiple flightlines in a single geographic area and for scenes across sites. Analysis of data from multiple flightlines requires consistent implementation of atmospheric correction, but bidirectional reflectance distribution function (BRDF) effects also strongly influence brightness across images. In studies where multiple flightlines must be mosaicked for analysis or where retrievals from across multiple flightlines are to be compared, cross‐track brightness gradients due to BRDF may significantly confound analyses.

**Figure 1:**
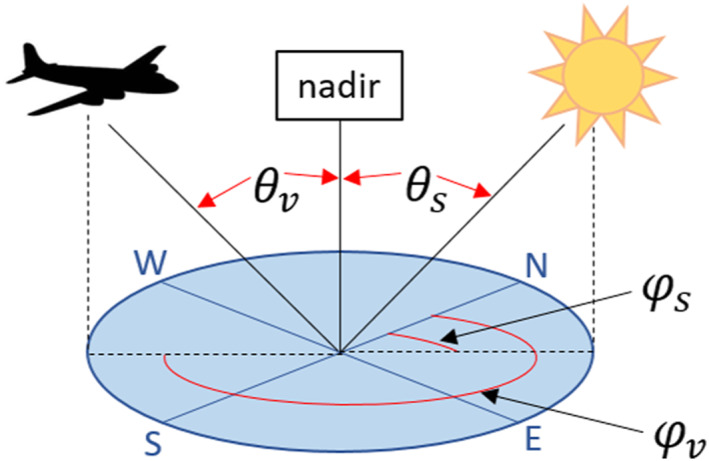
The bidirectional reflectance distribution function is a function of view zenith angle (*θ*_*v*_), solar zenith angle (*θ*_*s*_), and relative azimuth angle between sun and sensor (*φ* = *φ*_*s*_−*φ*_*v*_).

**Figure 2:**
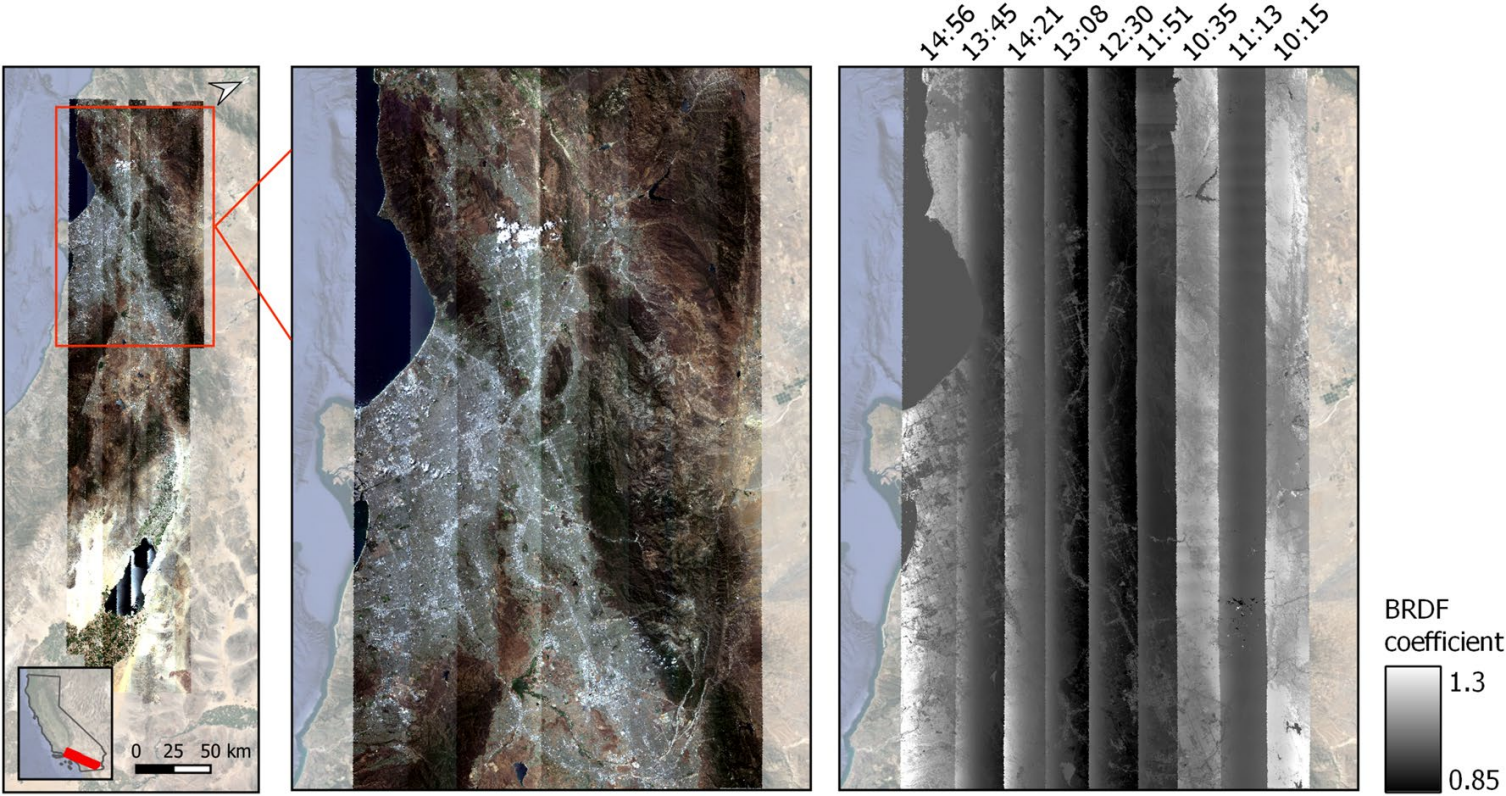
Bidirectional reflectance distribution function (BRDF) effects manifest as exceptional brightness and darkness on either end of an image. The middle panel shows an uncorrected, true‐color mosaic. For illustration, the right panel shows BRDF correction coefficients derived in this paper for a sample band (660 nm). Flightlines are labeled by their local acquisition time. The full flight box is shown on the left.

BRDF describes the reflectance of a surface by incoming and outgoing light. The function is parameterized by the zenith and azimuth angles of the incoming (solar) and outgoing (sensor) directions (Figure 1). The BRDF effects in imagery result from different sunlit/shaded portions of the same surface target seen by the sensor under different solar and view geometries (Nicodemus et al., 1977; Schaepman‐Strub et al., 2006). BRDF effects are most apparent on the seams between adjacent images and are manifested as increased brightness on one side of the flightline relative to the other due to sun‐sensor‐target geometry. BRDF effects also cause a difference in the relative brightness of the gradient across sets of images as a result of differing illumination conditions throughout the day. Both variations in brightness are evident in Figure 2. BRDF correction minimizes such effects by normalizing the reflectance to the same solar and view geometry. Naturally, this same solar and view geometry includes a constant view zenith angle (*θ*_*v*_) and solar zenith angle (*θ*_*s*_). This work was motivated by the observation that many BRDF correction approaches for airborne hyperspectral imagery sufficiently address the within‐flightline brightness gradients (i.e., correcting to nadir view) without addressing between‐flightline variations (Collings et al., 2010; Jensen et al., 2018; Jia et al., 2020), which may yield undesirable artifacts in subsequent mosaics or derived products. As such, it is important to ensure that the corrections further account for differences associated with images collected with differing solar geometries.

Here we present a flexible, automated approach to BRDF correction optimized for vegetation called flexible bidirectional reflectance distribution function (FlexBRDF) that is suitable for correcting multiple adjacent flightlines based on the combined sun‐sensor‐target reflectance characteristics across all lines in a flight box acquired on the same day. To minimize shadowing effects from steep terrain, all testing was conducted on topographically corrected reflectance data using the sun‐canopy‐sensor (SCS) + *C* (SCS + *C*) topographic correction method outlined in Soenen et al. (2005). FlexBRDF is built upon the Ross‐Li kernel‐driven semi‐empirical BRDF correction method, which implements a set of kernels approximated from physical models to describe different BRDF shapes (Wanner et al., 1995). The BRDF model is a linear combination of geometric, volumetric, and isotropic terms. Collectively, these terms incorporate the geometric structure of reflectors and shadowing effects, the volumetric scattering across randomly distributed facets (such as leaves), and the isotropic scattering contribution (i.e., uniform scattering in all directions; Roujean et al., 1992). This method is widely used for BRDF correction of airborne imaging spectroscopy data (Colgan et al., 2012; Collings et al., 2010; Schlapfer et al., 2015; Weyermann et al., 2015). To make a widely accessible BRDF correction that addresses within and across image brightness differences and is suitable for diverse land cover types and different sensors, we modify the basic kernel‐based method and allow for user customization.

Within‐image BRDF effects are caused by solar and view geometries and their interaction with land cover type (Nicodemus et al., 1977; Schaepman‐Strub et al., 2006), which influences the geometric and volumetric terms in the BRDF model. To address this, BRDF correction methods commonly use a pre‐classification scheme to stratify the BRDF correction by cover type with assumed similarities in crown structure (Colgan et al., 2012; Jensen et al., 2018; Jia et al., 2020; Weyermann et al., 2015), but this is not practical across large aerial campaigns covering a wide range of vegetation types that may be imaged at different spatial resolutions. BRDF Effects Correction for Wide‐Field‐of‐View Optical Scanners, described in Schläpfer et al. (2015), avoids the use of an ancillary land cover data set by stratifying images using a continuous BRDF cover index (BCI) derived from the normalized difference vegetation index (NDVI) and empirically derived adjustment factors. The benefit to an index such as BCI or NDVI over a pre‐classification for stratification is that it is continuous (i.e., pixels with similar index values are more likely to be alike than not) and, as a ratio, reduces influence of BRDF (Buchhorn et al., 2016) for stratification compared to pre‐classification, which would be performed on uncorrected imagery. It also enables interpolation of BRDF coefficients between stratification bins, which is not possible using a discrete pre‐classification approach. We chose to use NDVI for FlexBRDF because it is widely understood and employed in analysis of vegetation with remote sensing. Here, we test a range of approaches to NDVI binning, including a dynamic binning strategy that provides flexibility for flightlines of varying land cover heterogeneity.

Flightlines collected within a flight box can cover a range of illumination conditions and vegetation types across the solar window of acquisitions, such that long acquisition flights in areas with high landscape variation may not exhibit consistent BRDF corrections for individually corrected lines. FlexBRDF addresses the across‐image brightness variation in two ways. First, FlexBRDF corrects an entire flight box as a group rather than on a flightline‐by‐flightline basis, as in Colgan et al. (2012) and Schläpfer et al. (2015). Second, FlexBRDF accounts for the variation in solar and view geometries as data within a flight box are collected over several hours by providing a range of options to select a reference solar zenith angle for the BRDF correction, which is likewise necessary when integrating analyses from flightlines collected under widely varying illumination conditions (Chen et al., 1999; Colgan et al., 2012; Weyermann et al., 2015).

We demonstrate the generality of FlexBRDF across complex landscapes including Mediterranean, temperate, and Arctic biomes using airborne imaging spectroscopy data from NASA's 224‐band whiskbroom airborne visible/infrared imaging spectrometer (AVIRIS)‐Classic (Green et al., 1998) and data from several nearly identical 480‐band pushbroom sensors including AVIRIS‐Next Generation (NG; Hamlin et al., 2011) and the NEON Airborne Observation Platform (AOP; Kampe, 2010). These instruments image the visible to shortwave infrared spectrum (∼380–2,500 nm) with ±17–18° field of view. These sensors typically collect several long, narrow image strips (referred to as flightlines) for a given target area (referred to as a flight box). FlexBRDF was initially developed for a Southern California flight box and applied to subsequent boxes in India, Wisconsin, Alaska, and Yosemite, CA with favorable results. FlexBRDF is open‐source and available from GitHub as part of our HyTools toolkit, and has numerous options for topographic correction method, BRDF kernel combinations, vegetation index, binning strategy, and choice of reference solar zenith angle.

## 2 Study Area and Data

We initially developed FlexBRDF using imagery collected on 22 May 2013 by AVIRIS‐Classic for a Southern California flight box that has been routinely flown ‐ sometimes multiple times per year ‐ since 2013 (Lee et al., 2015). Images from this flight box exhibit significant brightness differences across flightlines (Figure 2). In addition, this flight box presents multiple challenges requiring a robust BRDF correction approach: Very long flightlines collected across five hours spanning a range of illumination angles, as well as diverse vegetation and considerable topography. The box stretches from the Pacific ocean to the Salton sea, with a range of elevation spanning −70–3,068 m asl., and contains prominent geological features such as the Santa Monica and San Gabriel mountains, as well as the densely populated city of Los Angeles. Atmospherically corrected surface reflectance data were accessed through the AVIRIS data portal (https://aviris.jpl.nasa.gov/dataportal/). We used nine overlapping flightlines, with spatial resolutions ranging from 14.4 to 15.8 m, and a spectral resolution of 10 nm at 224 wavebands from 400 to 2,500 nm (Green et al., 1998). For AVIRIS‐Classic, FlexBRDF used L1B observation geometry data (specifically, files labeled obs_ort) and L2 surface reflectance images that had been atmospherically corrected using ATREM (Thompson et al., 2015).

We tested FlexBRDF and refined our code using data acquired from several different sensors over different land cover types (Table 1). The testing was designed to assess the generality of the approach, using sites with significant heterogeneity (Southern California, Yosemite, Fairbanks), moderate variation (Mudumalai) and comparatively homogeneous land cover (Wisconsin). Application of an automated approach to correcting NEON data in particular is necessary, as NEON flies dozens of sites annually using multiple imaging spectrometer payloads, generating over 1000 flightlines per year. Likewise, data from California, India, and Alaska were all collected by NASA in support of large airborne campaigns for which single‐line corrections are both impractical and likely to generate highly variable results. All analyses use atmospherically corrected reflectance imagery as provided by the data distributor. “Uncorrected” reflectance will henceforth refer to these products prior to the application of topographic and BRDF corrections.

**Table 1:** Sensor and Environmental Properties of All Test Sites

Flight box	Date of acquisition	Sensor	Spatial res (m)	Spectral res (nm)	# Bands	Land cover
Southern California 2013	22 May 2013	AVIRIS‐C	14.4–15.8	10	224	Urban, chaparral, oak woodland, conifer forest
Southern California 2016	16 June 2016	AVIRIS‐C	14.4–15.6	10	224	Urban, chaparral, oak woodland, conifer forest
Yosemite, CA	7 June 2017	AVIRIS‐C	13.7–14.9	10	224	Conifer forest, woodland, snow
Fairbanks, AK	23 July 2018	AVIRIS‐NG	5.2	5	425	Boreal forest
Mudumalai, India	5 January 2016	AVIRIS‐NG	4	5	425	Subtropical and tropical forest
Chequamegon, WI	11 September 2017	NEON	1	5	425	Hardwood and conifer forest

*Note*. See Table S1 for list of specific flightlines used. AVIRIS, airborne visible/infrared imaging spectrometer.

## 3 Methods

### 3.1 Topographic Correction

In areas with complex terrain, a topographic correction should be implemented prior to or in conjunction with the BRDF correction (Jia et al., 2020; Schläpfer et al., 2015; Weyermann et al., 2015), since terrain orientation in relation to solar and view angles affects pixel brightness (Justice et al., 1981). We corrected for topography on a flightline‐by‐flightline basis, following the SCS with *C*‐correction (SCS + *C*) described by Soenen et al. (2005). The SCS + *C* correction combines the *C*‐correction, which assumes consistent geometry of terrain and trees, with normalized SCS geometry. Topography‐induced brightness difference is corrected for each pixel as a function of its slope, solar zenith angle, and relative azimuth angle between the sun and terrain (*φ*_*t*_).

(1) $$R_{t}=R\frac{\cos\;\alpha \;\cos\;\theta_{s}+C}{\cos\;i+C},$$

where *R*_*t*_ is topographically corrected reflectance, *R* is uncorrected reflectance, *α* is the terrain slope, and cos *i* is the illumination factor, calculated as:

(2) $$\cos\;i=\cos\;\alpha \;\cos\;\theta_{s}+\sin\;\alpha \;\sin\;\theta_{s}\;\cos\;\varphi_{t}$$

*C* is a function of the parameters of the linear relationship observed between *R* and *cos* *i*, shown in Equation 3.

(3) R=a+bcosiandC=a/b

### 3.2 Kernels

FlexBRDF uses the Ross and Li kernels, which approximate BRDF shapes at varying solar and view zenith, and azimuth angles. The Li geometric kernels describe the arrangement of objects; Li‐Sparse refers to sparse vegetation with characteristically prominent shadowing and Li‐Dense refers to dense vegetation. The Ross volumetric kernels describe the distribution of facets (i.e., leaves); Ross‐Thick assumes LAI ≥ 1, while Ross‐Thin assumes LAI < 1. The kernel‐based method is a semi‐empirical approach, chosen for its computational feasibility, ability to scale spatially, and because it accounts for subpixel heterogeneity in land cover.

We tested four kernel combinations (sparse‐thick, sparse‐thin, dense‐thick, dense‐thin) to identify the set of kernels to apply. Our objective was not to thoroughly test the kernels but rather to identify the kernel combinations that produced consistent corrections across our diverse landscapes, namely reduction of the across‐track brightness effect. Illustrated graphically, Li‐Dense kernels resulted in overcorrection of pixels in the forward scattering direction in comparison to the Li‐Sparse kernel (Figure S1 in Supporting Information S1), while the difference between thick and thin was negligible. Subsequent implementation exclusively used the Ross‐Thick and Li‐Sparse kernels, which are widely used in other relevant studies (Schläpfer et al., 2015; Weyermann et al., 2015), and seem to perform most consistently across a wide range of surface conditions. The open source code for our method allows users to select a range of kernels appropriate to their application. Full mathematical formulation for the derivation of the selected geometric and volumetric kernels (*K*_geo_ and *K*_vol_) follows Wanner et al. (1995):

(4a) $$\theta^{\prime }=\tan^{-1}\left(\frac{b}{r}\;\tan\theta \right)$$

(4b) $$D=\sqrt{tan^{2}\;\theta_{s}^{\prime }+tan^{2}\;\theta_{v}^{\prime }-2\;\tan\;\theta_{s}^{\prime }\;\tan\;\theta_{v}^{\prime }\;\cos\;\varphi }$$

(4c) $$\cos\;t=\frac{h}{b}\frac{\sqrt{D^{2}+{\left(\tan\;\theta_{s}^{\prime }\;\tan\;\theta_{v}^{\prime }\;\sin\;\varphi \right)}^{2}}}{\sec\;\theta_{s}^{\prime }+\sec\;\theta_{v}^{\prime }}$$

(4d) $$O=\frac{1}{\pi }\left(t-\sin\;t\;\cos\;t\right)\left(\sec\;\theta_{s}^{\prime }+\sec\;\theta_{v}^{\prime }\right)$$

(4e) $$\cos\;\xi^{\prime }=\cos\;\theta_{s}^{\prime }\;\cos\;\theta_{v}^{\prime }+\sin\;\theta_{s}^{\prime }\;\sin\;\theta_{v}^{\prime }\;\cos\;\varphi$$

(4f) $$K_{\text{geo}}=O(\theta_{s},\;\theta_{v},\;\varphi )-\sec\;\theta_{s}^{\prime }-\sec\;\theta_{v}^{\prime }+\frac{1}{2}\;(1+\cos\;\xi^{\prime })\;\sec\;\theta_{v}^{\prime }$$

(4g) $$K_{\text{vol}}=\frac{(\pi /2-\xi )\cos\;\xi +\sin\;\xi }{\cos\;\theta_{s}+\cos\;\theta_{v}}-\frac{\pi }{4}\;\xi ,$$

where the fractions *b*/*r* and *h*/*b* are constants describing vegetation shape and height. In our case, we use the constant values (10 and 2, respectively) derived in Colgan et al. (2012).

### 3.3 BRDF Correction Model

BRDF is calculated as follows per wavelength and per pixel:

(5) $$\rho \left(\theta_{v},\theta_{s},\varphi \right)=f_{\text{iso}}+f_{\text{geo}}K_{\text{geo}}\left(\theta_{v},\theta_{s},\varphi \right)+f_{\text{vol}}K_{\text{vol}}\left(\theta_{v},\theta_{s},\varphi \right)$$

**Figure 3:**
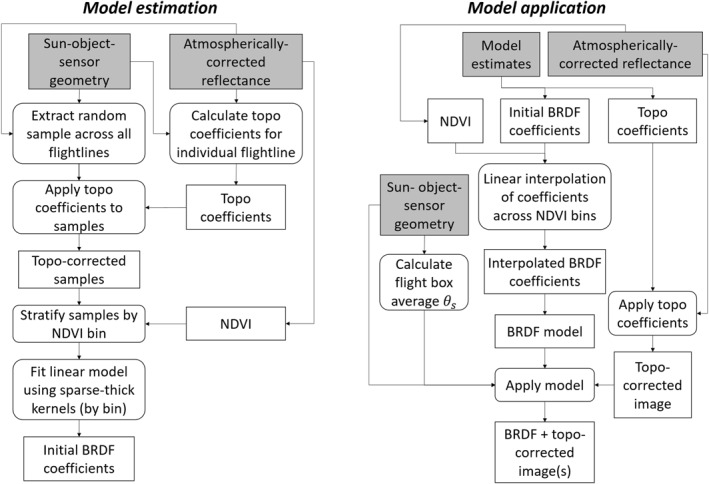
A flexible bidirectional reflectance distribution function model estimation and application workflows.

The *K* parameters are the kernel values, which are fixed values derived for each wavelength and are a function of each pixel's combination of view and solar angles. The *f* values are the least squares regression coefficients (referred to here as BRDF coefficients) that account for varying vegetation structure in the sampled pixels. The set of geometric, volumetric, and isotropic weighting coefficients‐*f*_iso_, *f*_geo_, *f*_vol_‐describe a characteristic land cover type. For this reason, BRDF corrections generally use a pre‐classification scheme (e.g., Jensen et al. [2018]) or alternatively an index (e.g., Schläpfer et al. [2015]) or, in our case, NDVI binning. The full model estimation workflow is shown in Figure 3.

The BRDF correction approximates nadir (*θ*_*v*_ = 0) reflectance for each pixel based on the calculated kernel values and derived BRDF coefficients. When *θ*_*v*_ = 0, *φ*_*v*_ becomes arbitrary. As such, we use a relative azimuth angle that is normalized to an arbitrary number (*φ*_*n*_). Equation 6 describes the application of the correction factors, which compare the observed reflectance of a pixel (or, in this case, the topographically corrected reflectance *R*_*t*_) to the modeled reflectance at nadir. These factors are calculated and applied on a per‐wavelength and per‐pixel basis, outputting topographic‐ and BRDF‐corrected reflectance *R*_tb_.

(6) $$R_{\text{tb}}\;=\;R_{t}\left(0,\theta_{s},\varphi_{n}\right)=R_{t}\left(\theta_{v},\theta_{s},\varphi \right)\frac{\rho \left(0,\theta_{s},\varphi_{n}\right)}{\rho \left(\theta_{v},\theta_{s},\varphi \right)}$$

### 3.4 NDVI Stratification

Pre‐correction stratification of imagery is often used in BRDF correction to ensure that correction coefficients capture differences in vegetation canopy characteristics (e.g., structure, leaf shape, leaf area, etc.; Colgan et al., 2012; Jensen et al., 2018; Jia et al., 2020; Weyermann et al., 2015). Rather than a discrete classification scheme, we use a vegetation index (Schläpfer et al., 2015), which can facilitate a continuous stratification of vegetation. Reflectance image pixels were binned based on NDVI calculated as (*R*_850_ − *R*_665_)/(*R*_850_ + *R*_665_). BRDF coefficients were initially calculated for each bin separately, so that each bin was corrected by a constant value. To assess the effect of NDVI bin size on the corrected results, we ran BRDF corrections with 3, 8, and 18 bins for values of NDVI >0.05. Once binned, BRDF coefficients were calculated using Equation 5 for each NDVI bin separately.

Initial tests used static predefined bins (Table S2) in which we masked out pixels with very low NDVI values (<0.05) to account for water, non‐vegetation, and anomalous values. For output maps, original reflectance values were retained in the BRDF‐corrected product in masked areas. After initial testing, we also implemented a dynamic binning approach that utilized 18 bins with divisions of equal sample size within the range of 0.05 < NDVI <0.9. This approach aimed to accommodate images in which NDVI variability was within a narrow range, such as in the NEON Wisconsin box used in our study (Figure S2 in Supporting Information S1).

Like a cover type classification, there is the risk of abrupt boundaries in BRDF‐corrected reflectance at transitions between NDVI bins. To prevent edge effects between NDVI classes in the corrected imagery, we tested smoothing techniques between bins for the weighting factors using the most numerous bin approach (18‐bin). As a result, BRDF coefficients are regressed or interpolated to be continuous across the NDVI bins rather than constant values for each bin. We tested: unweighted linear regression (where coefficients are modeled as a function of NDVI), weighted linear regression (based on the size of the bin), and linear interpolation using the Python SciPy library (Virtanen et al., 2020). To avoid overcorrection in very low (0.05–0.25) and very high (0.85–1) NDVI, smoothing methods were applied only on bins between 0.25 and 0.85. Although we only compared corrections using NDVI bins, our open‐source code can accommodate a user‐supplied classification or use an alternate continuous index to NDVI.

### 3.5 Reference Solar Zenith Angle

In general, correcting BRDF for a single flightline models nadir‐viewing reflectance (*θ*_*v*_ = 0) for each pixel based on the solar zenith angle (*θ*_*s*_) at the time of flight. For flight boxes in which lines are acquired over multiple hours within a day, this correction approach introduces systematic line‐to‐line brightness differences. FlexBRDF thus corrects all lines within a flight box to a reference solar zenith angle (*θ*_*sr*_; Chen et al., 1999; Colgan et al., 2012; Weyermann et al., 2015). As such, *θ*_*s*_ can be substituted for *θ*_*sr*_ in Equation 6 to output topographic‐ and BRDF‐corrected reflectance with solar zenith reference *R*_*tbz*_ (Equation 7). The full model application workflow is shown in Figure 3.

(7) $$R_{\text{tbz}}=R_{t}\left(0,\theta_{sr},\varphi_{n}\right)=R_{t}\left(\theta_{v},\theta_{s},\varphi \right)\frac{\rho \left(0,\theta_{sr},\varphi_{n}\right)}{\rho \left(\theta_{v},\theta_{s},\varphi \right)}$$

The selection of *θ*_*sr*_ for a flight box is not straightforward, especially if analyses of images from one date are to be integrated with data from images collected on another date. To assess the ramifications, we compared four values of *θ*_*sr*_ (Table 2) in our implementation of FlexBRDF for the initial 22 May 2013 Southern California flight box using the 18‐bin group with linear interpolation. For all tests, we used the latitude (33.7°) averaged from the center points of all images to determine *θ*_*sr*_. Approach *θ*_*s*1_ uses the solar zenith angle at solar noon on the date of acquisition, which could reasonably be considered a default approach for some applications. *θ*_*s*2_ uses the average of all solar zenith angles at solar noon across the date range encompassing the growing season, which can be seen as a generalized value suitable for integrating data from multiple dates but risks overcorrecting images with solar zenith angles that differ substantially from the seasonal average (Zhang et al., 2016). *θ*_*s*3_ uses the average observed *θ*_*s*_ from the flight box, which could be considered a relatively cautious default, but could have implications for integrating images across dates. *θ*_*s*4_ uses the solar zenith angle at solar noon on the summer solstice, which might also be considered a reasonable baseline for integrating data from across the growing season, but will also always be a value that is at the extreme of observed values for any given flight box. Of note, any approach that uses *θ*_*sr*_ tied to solar noon can be problematic if all flightlines are collected before or after noon.

**Table 2:** Solar Zenith Angles in Degrees From Nadir Tested for Bidirectional Reflectance Distribution Function Correction

Name	Calculation	Solar zenith angle
*θ*_*s*1_	Solar noon on day of acquisition	13.78
*θ*_*s*2_	Solar noon averaged for growing season	22.22 (April–October) 14.59 (June–August)
*θ*_*s*3_	Observed average *θ*_*s*_ for all lines in flight box	23.87
*θ*_*s*4_	Solar noon on summer solstice	10.37

*Note*. Values are provided for the 22 May 2013 image used for initial development of the method. Two periods were tested for *θ*_*s*2_: A broad estimate for growing season (April–October) and a more restricted period (June–August). The April–October period was closer to the flight box average, and is shown in subsequent results.

### 3.6 Assessment

First, we qualitatively evaluated spectral continuity across flightlines by inspecting for clear boundaries between images within a flight box or between NDVI stratification classes within an image, both of which would indicate a poor correction. We further assessed spectral continuity across the flight box through visual inspection of spectra and transects. Spectra were drawn from corresponding vegetation and soil ROIs in one overlapping area with relatively prominent BRDF effects in Southern California. Spectra were averaged for each ROI. Four transects were drawn across the flight box, oriented perpendicular to the rotation angle of the images (−67° for the California test images). Points were distributed every 500 m across each transect. For each point, we calculated the mean reflectance value from a 5 pixel by 5 pixel window to account for possible errors in image registration. We plotted transects for eight bands covering the portion of the VSWIR range that most prominently exhibits BRDF effects (480, 560, 660, 850, 975, 1,050, 1,150, and 1,240 nm). Discontinuities in reflectance values at image boundaries across a transect indicate the presence of residual BRDF effects.

Quantitatively, we performed a direct comparison of pixel values between overlapping areas of adjacent flightlines to assess spectral consistency. Due to imperfect spatial registration, individual pixels from overlapping areas do not necessarily align. To address this, we performed co‐registration between the overlapping areas using spectral matching (see Text S1 in Supporting Information S1 for details). For the May 2013 Southern California box, we extracted the overlapping pixels for each wavelength of the entire image spectrum, ran a simple linear regression, and calculated the root mean square error (RMSE) and mean absolute deviation (MAD) following Jensen et al. (2018) and Collings et al. (2010). Lower RMSE and MAD indicate a closer match between overlapping areas, and are thus interpreted as a better correction. These metrics were averaged across all overlap areas in a flight box by wavelength. We repeated the method for subsequent test flight boxes using a 10‐band spectral subset (480, 560, 660, 850, 975, 1,050, 1,150, 1,240, 1,650, and 2,215 nm) instead of the full spectrum.

## 4 Results

### 4.1 Southern California 2013

**Figure 4:**
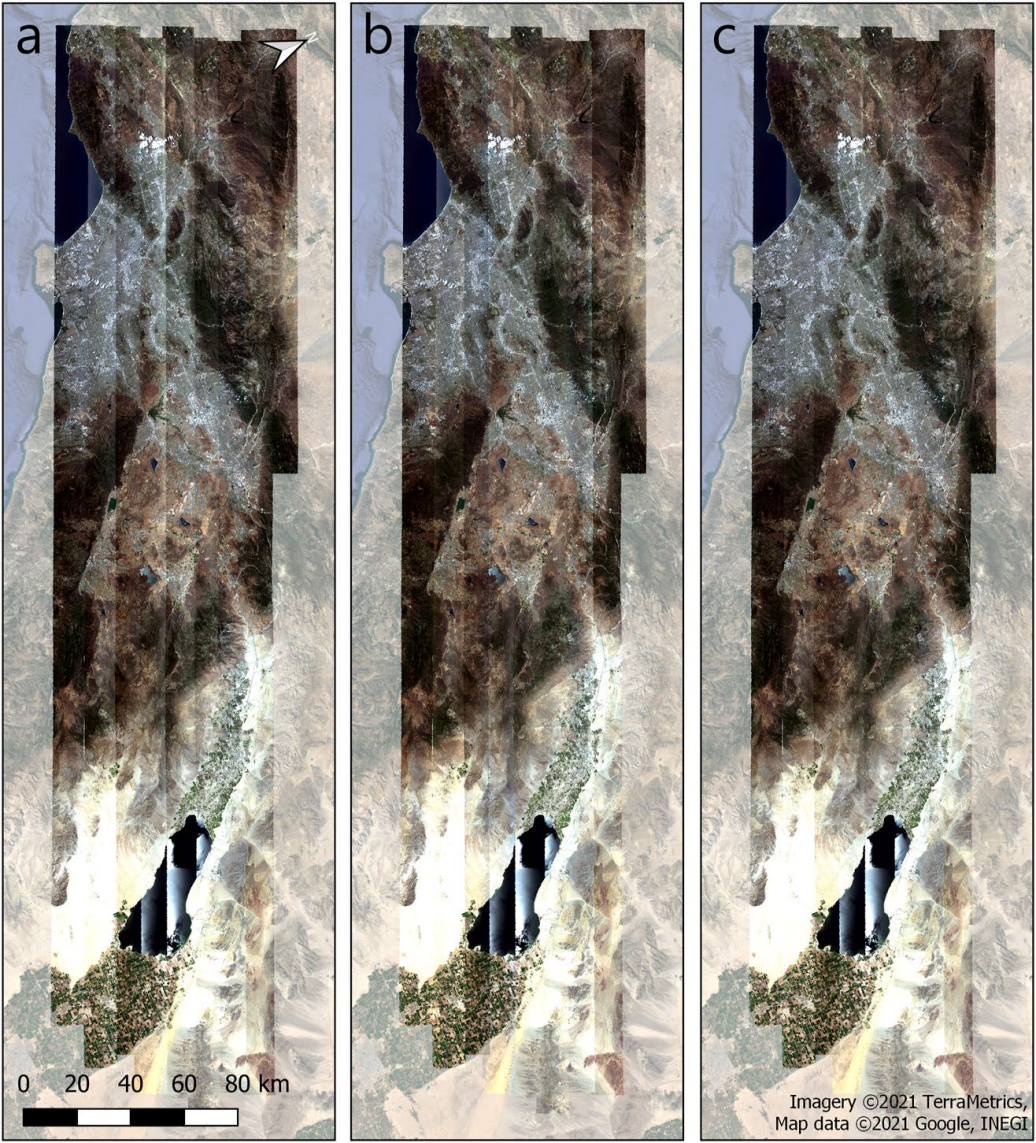
Mosaicked true color reflectance images over Southern California, showing (a) no bidirectional reflectance distribution function correction, (b) 18‐bin single correction, and (c) 18‐bin group correction.

**Figure 5:**
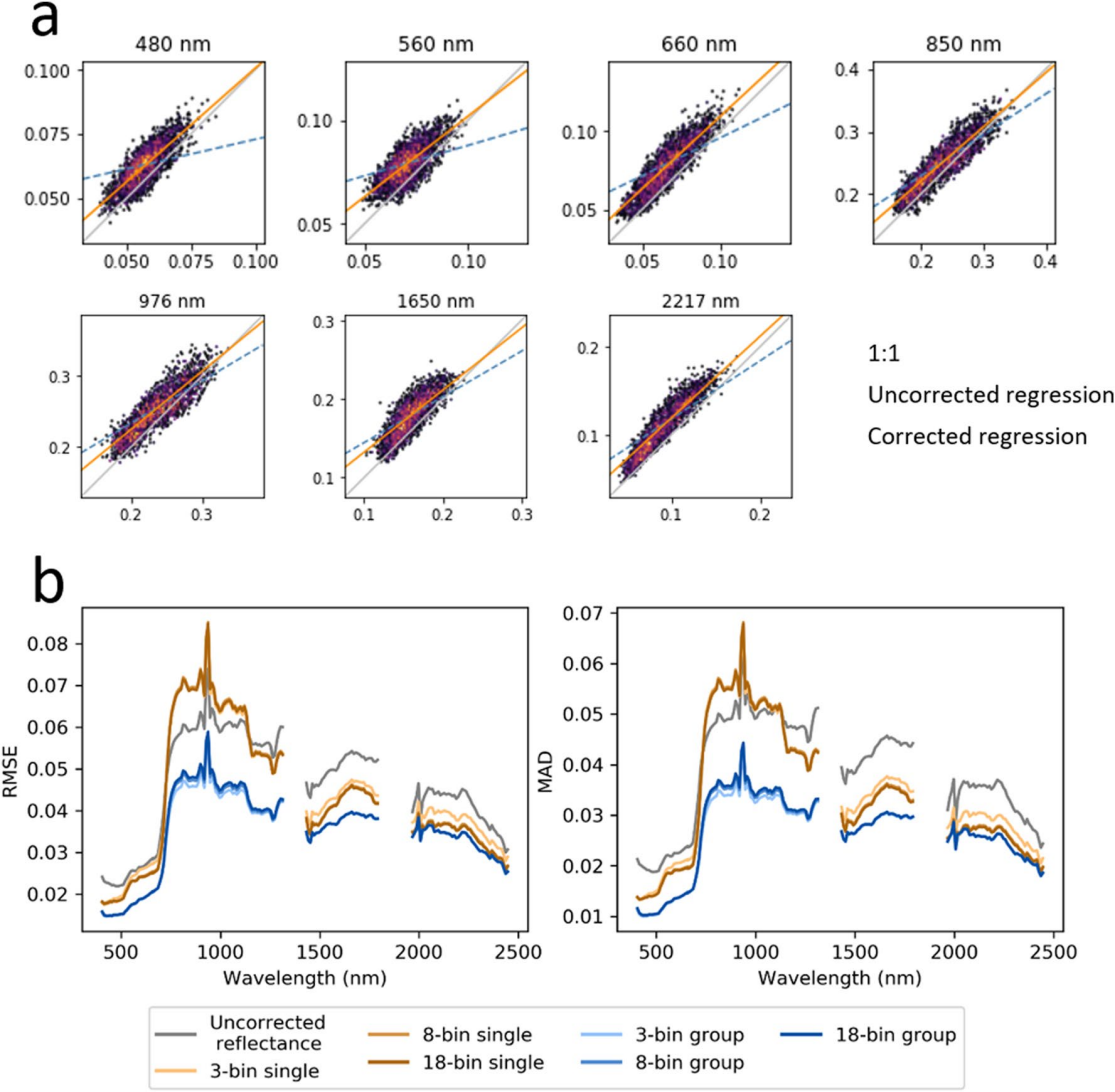
Scatterplots (a) show overlapping reflectance for two flightlines from the 2013 Southern California flight box. Regression lines are shown for overlapping areas in uncorrected (dashed blue, points not shown) and corrected (orange, points shown) imagery. Perfect spectral consistency would show points falling along the 1:1 line (gray). (b) Root mean square error and mean absolute deviation from the simple linear regression fit are averaged for all overlapping areas in the flight box and shown for the entire spectrum.

To determine our optimal FlexBRDF parameterization, we first compared the performance of group processing to single line processing. Qualitatively, group processing yielded a mosaic with minimized seamlines between adjacent flightlines, while single line processing did not eliminate all seamlines (Figure 4). Quantitatively, all group processing iterations yielded lower RMSE (up to 2.7% less, varying by wavelength) and MAD (up to 2.4% less) in overlap areas than single line processing and uncorrected reflectance (Figure 5; Table S3 in Supporting Information S1). In contrast, all three single processing iterations (3‐bin, 8‐bin, and 18‐bin) yielded higher RMSE (up to 1.1% higher) and MAD (up to 0.7% higher) than the original reflectance in the red edge/NIR range.

We compared the effect of bin size on model performance for both single line and group corrections. Grouping clearly had a larger effect on model performance than bin size, as evident in differences in RMSE and MAD in Figure 5. Therefore, we focus on the bin size results for the grouped method only. The various grouping methods performed similarly across most of the spectrum, but the 3‐bin approach yielded the lowest values for RMSE and MAD in the 700–1,400 nm range. Qualitatively, the number of NDVI bins did not affect the overall appearance of boundaries between flightlines, but the use of fewer bins resulted in more prominent edges between NDVI classes in the corrected image (and conversely, more bins minimized between‐bin edges; Figure S3 in Supporting Information S1). In the use of static bins, we found that smaller and more numerous NDVI bins produced corrections with less evidence of edges among classes or between flightlines. Therefore, further results will focus on 18‐bin methods.

In pursuing minimized between‐bin boundaries, we assessed the inclusion of a BRDF coefficient smoothing method for the 18‐bin grouped approach (Figure S4 in Supporting Information S1). We tested linear regression, weighted linear regression, and linear interpolation of BRDF coefficients, and found that the inclusion of a smoothing method resulted in comparable RMSE and MAD values to unsmoothed coefficients, but reduced visual discontinuities across NDVI class boundaries. Of the different smoothing methods, the weighted linear regression is the only method that performed noticeably worse across the entire spectrum, especially in the 2,000–2,500 nm range (Figure S5 in Supporting Information S1). Because linear interpolation and linear regression performed similarly, and linear interpolation better preserves the shape of the BRDF coefficients across NDVI bins, further results will focus on methods with linear interpolation.

**Figure 6:**
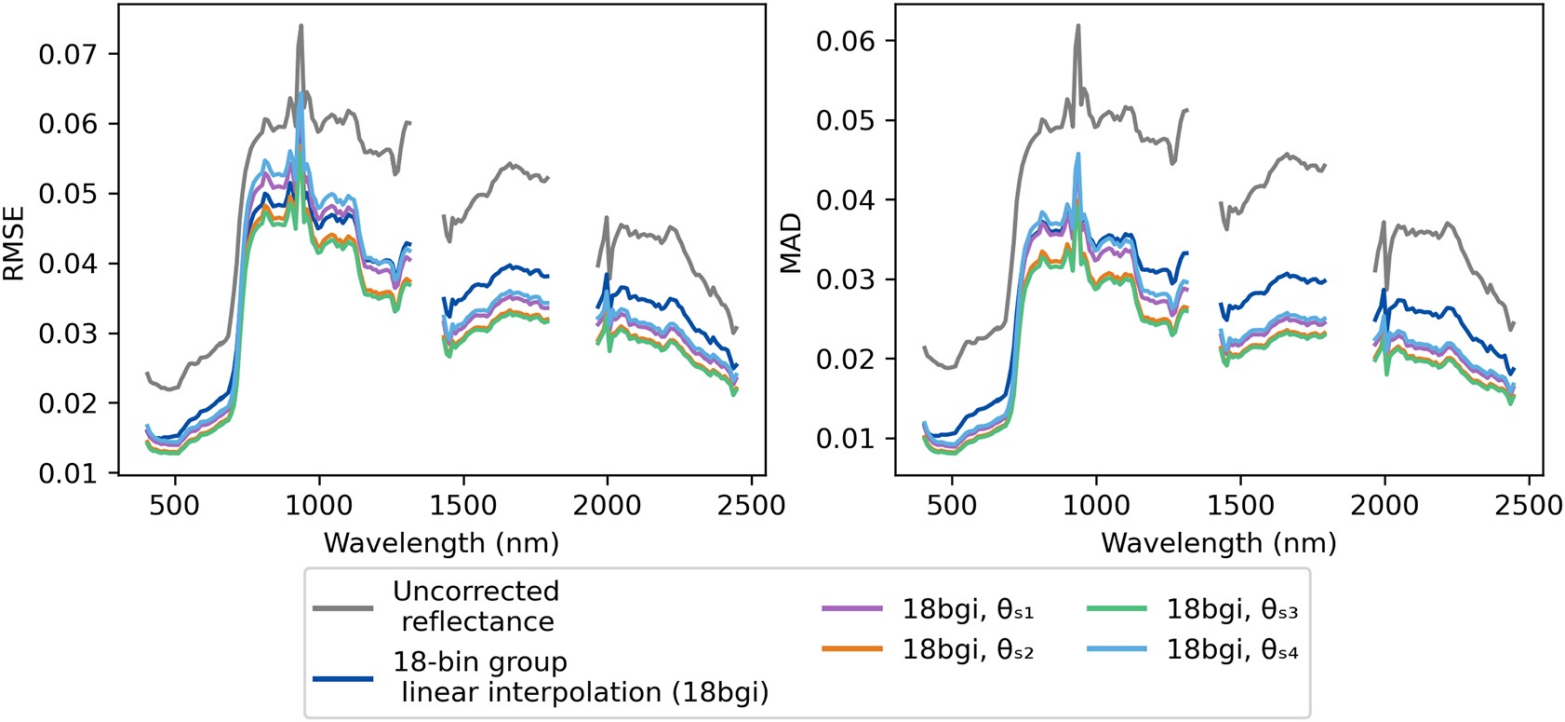
Root mean square error and mean absolute deviation of overlapping pixels shown for all four reference solar zenith angle tests compared to uncorrected reflectance and corrected reflectance with no solar zenith reference.

Finally, we tested the four options for *θ*_*sr*_ outlined in Table 2 to further address between‐line brightness variation. The inclusion of *θ*_*sr*_ improved spectral consistency across the flight box, both visually and quantitatively. While all four test angles yielded visually smooth mosaics, *θ*_*s*2_ and *θ*_*s*3_ resulted in comparably low RMSE and MAD values (up to 0.7% lower than correction with no *θ*_*sr*_; Figure 6). *θ*_*s*3_ was chosen for the finalized method, which will be further discussed in the discussion section.

**Figure 7:**
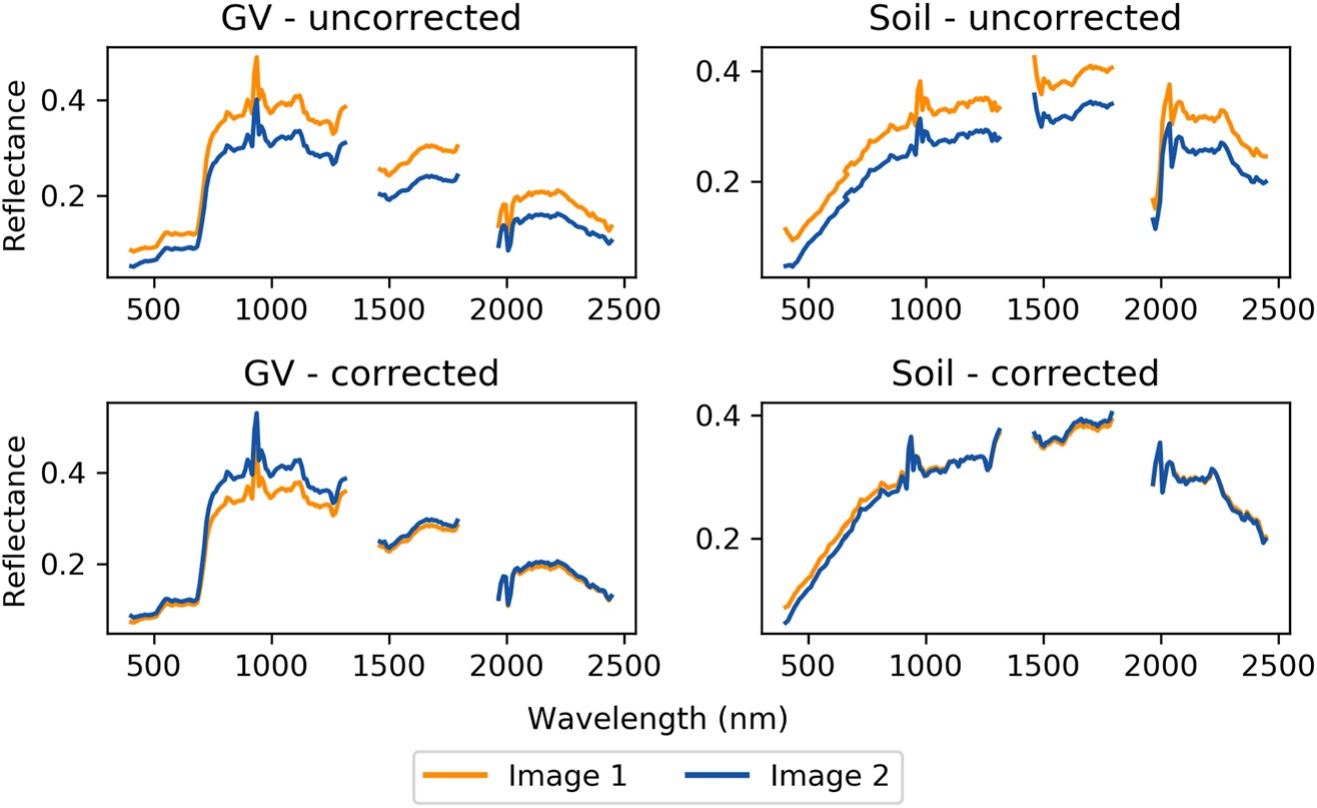
Averaged spectra shown for overlapping green vegetation and soil patches in both uncorrected and bidirectional reflectance distribution function‐corrected imagery.

The finalized parameterization, which used group processing, 18 NDVI bins with linear interpolation, and *θ*_*s*3_, yielded a visually smooth mosaic with favorably low assessment metrics as compared to the uncorrected imagery. Spectral data extracted from transects across the flightlines generally exhibited more continuous reflectance values in overlap areas within the final corrected images, or, at worse, minimal difference from uncorrected data (Figure S6 in Supporting Information S1). Spectra extracted from corresponding green vegetation and soil ROIs in overlapping areas appeared closer together in corrected images than uncorrected images (imagery with perfect atmospheric and BRDF correction would yield identical spectra; Figure 7). Root mean square error and MAD in corrected images improved up to 2.3% and 2.5%, varying by wavelength (Table S3 in Supporting Information S1).

### 4.2 Other Sites

**Figure 8:**
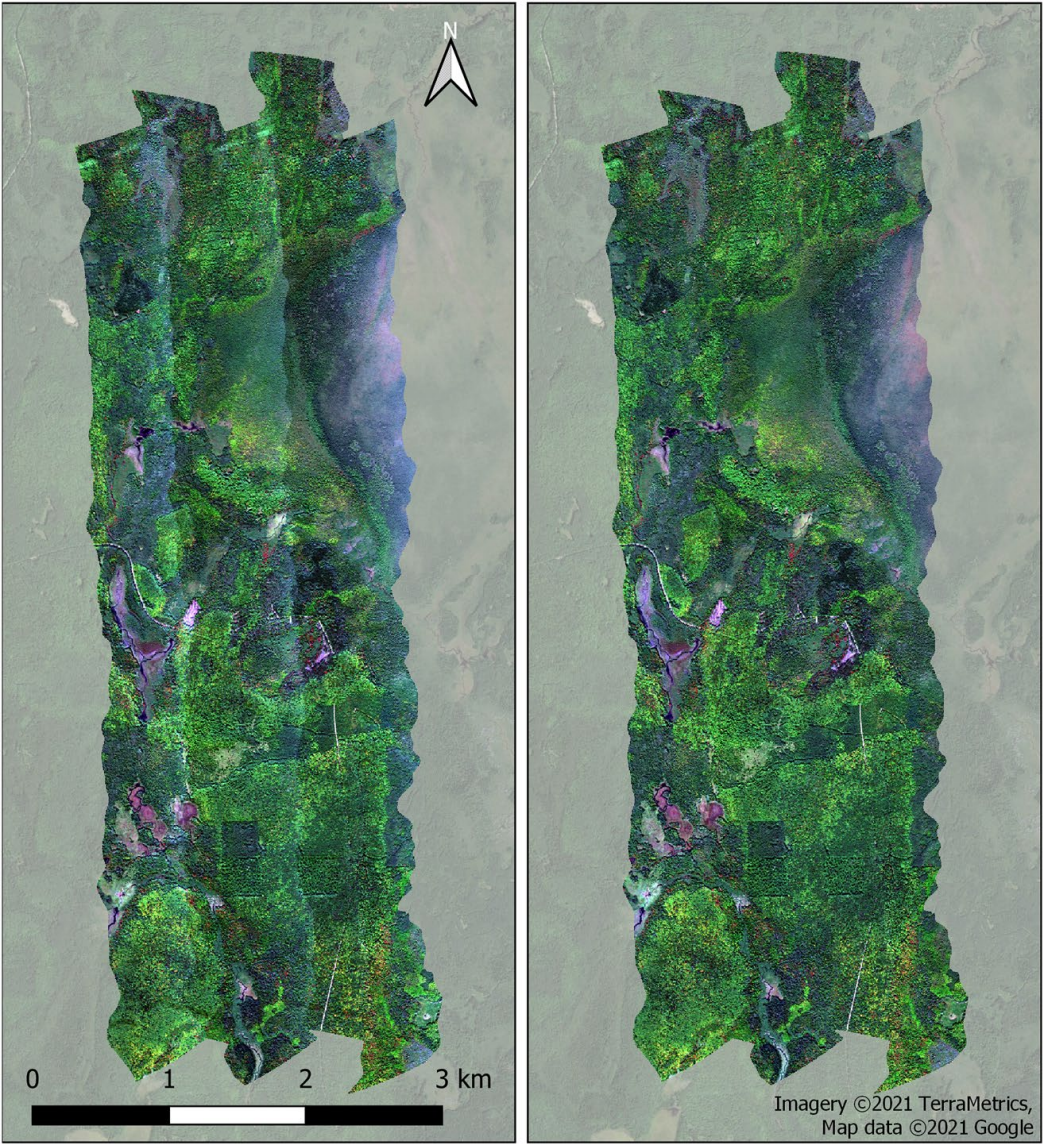
Before (left) and after (right) bidirectional reflectance distribution function correction for the U.S. National Ecological Observatory Network‐Wisconsin site. Other sites are shown in Figure S8 in Supporting Information S1.

For secondary test sites, we tested this optimized parameterization. Based on results from 22 May 2013 Southern California imagery, 18‐bin group with interpolation and *θ*_*s*3_ appeared to be the best option, but we tested both with and without the solar zenith angle standardization to see how its influence varied across flight boxes of different sizes with different illumination ranges across the data acquisition period. In all sites, both BRDF correction methods yielded lower RMSE and MAD than uncorrected reflectance (Figure S7 in Supporting Information S1), as well as a visually more continuous mosaic (Figure 8 and Figure S8 in Supporting Information S1). The inclusion of *θ*_*s*3_ had the largest effect on California boxes, lowering RMSE and MAD for the two Southern California boxes. For the Yosemite box, the inclusion of *θ*_*s*3_ performed worse than the 18‐bin without *θ*_*sr*_, though it still yielded lower RMSE and MAD than the uncorrected data. The inclusion of *θ*_*sr*_ made virtually no difference on RMSE and MAD of corrected imagery in the remaining boxes (Figure S7 in Supporting Information S1). The dynamic binning approach, which aimed to address the small range of NDVI values observed in the NEON Wisconsin box (further discussed below), had virtually no effect on RMSE and MAD (Figure S9 in Supporting Information S1), but is recommended for most applications because it lessens the chance for anomalous results if a fixed bin has a small sample size for computing BRDF coefficients.

## 5 Discussion

Grouping images for BRDF correction ensures a consistent correction across the flight box regardless of variation in solar illumination within and between flightlines. Models trained on single images do not account for the varying solar illumination observed across the entire flight box acquisition. This is likely why the 3, 8, and 18‐bin single corrections all yielded higher RMSE than uncorrected data in the 700–1,200 range (Figure 5). Based on differences in relative brightness across each image's brightness gradient, it is possible that two overlapping areas could be closer in reflectance before correction (as compared to single line correction) due to BRDF effects. Because the group correction draws a random sample across the entire box, the correction accounts for the varying illumination conditions experienced by the flightlines across several hours of data acquisition (see Figure S10 in Supporting Information S1).

Due to large flightline size and land cover complexity, we also suspect that some single image corrections are too heavily influenced by the proportion and geographic location within a flightline of certain land cover types, such as agriculture. Compared to single line processing, grouping of images improved consistency of the shape and pattern of BRDF coefficients by NDVI bin and across wavelengths. The isotropic coefficient in particular is a good indicator of BRDF model performance because it represents the bidirectional reflectance at *θ*_*v*_ = 0 for a given pixel (Roujean et al., 1992). For high NDVI pixels, the reflectance should resemble a vegetation spectrum while for low NDVI pixels, it should appear like a soil spectrum. The grouped method is able to produce physically sound isotropic coefficients (Figures S11c and S11i in Supporting Information S1) while the single line method yields inconsistent, and in some cases, unreasonable results (Figures S11f and S11m in Supporting Information S1).

Of the correction models we tested on the 22 May 2013 Southern California box, all grouping methods outperformed the single line methods and had a larger effect than NDVI bin size. While the different grouping methods all yielded similar results, the 3‐bin and 18‐bin grouping methods represented a trade‐off: By a small amount, the 3‐bin grouping exhibited the lowest RMSE and MAD values in overlap areas, while the 18‐bin grouping produced the most continuous output image, with less evidence of borders and only slightly higher MAD and RMSE. For this reason, we prefer 18 bins.

We expected that the NEON box would be a unique case for NDVI binning because of its constricted range of NDVI values, in which 90% of the NDVI values range between 0.7 and 0.9 (Figure S2 in Supporting Information S1). To avoid numerous NDVI bins with minimal samples for computing BRDF coefficients, we revised our static bin approach to create a dynamic binning approach, where the bins are defined by equal area of an NDVI bin rather than a fixed NDVI bin breadth. However, the test of an 18‐bin dynamic group correction yielded RMSE and MAD values that were virtually identical to the static binning (Figure S9 in Supporting Information S1). This suggests that either static binning or dynamic binning work well in homogeneous landscapes, but we expect that dynamic binning may have advantages in other homogenous situations. For example, dynamic binning may be advantageous in scenes where the NDVI range is largely restricted to lower values. This could be the basis for further testing: further development could test optimization approaches to dynamically vary the range of factors used in BRDF correction (e.g., including kernel selection).

Establishing a reference solar zenith angle is critical for highly diverse scenes and/or very long flightlines. Of the four angles we tested for the 22 May 2013 Southern California box, we achieved the best results for *θ*_*s*2_ and *θ*_*s*3_. The default selection we recommend is *θ*_*s*3_, since this is the most accurate representation of the average solar zenith angle observed on the day of data collection. The inclusion of a solar zenith reference angle was beneficial in the second Southern California flight box, but appeared detrimental for the Yosemite box and had negligible impact on the remaining sites (Figure S7 in Supporting Information S1). It is likely that Southern California saw the biggest improvement from the inclusion of a reference solar zenith angle because these lines are exceptionally long, and acquired over a span of many hours (and thus across a large range of solar zenith angles) in one day. Though we see favorable results for the complex scenes (Southern California), we recognize that adjustments to corrected reflectance as a consequence of using a common solar zenith angle could benefit from further study. If the user aims to compare flight boxes from the same location acquired on different dates, it may be useful to standardize these boxes to the same angle by setting one reference zenith angle for that particular box for all dates (Zhang et al., 2016). For that we recommend *θ*_*s*2_, which is determined based on the growing season average solar zenith angle for the flight box center. The caveat of *θ*_*s*2_ is that the user must choose the appropriate growing season range or another date range based on the context of the study.

All of our examples used groups of images that overlapped and were acquired on the same day. However, the method may be used on single images as well. Future work should test the extent to which grouping criteria can be extended or should be restricted. First, although it is reasonable to expect that this method would work on flightlines that are in close proximity but not overlapping, it would be difficult to assess model performance without overlapping areas. As well, it would be hard to specify a cutoff time beyond which images should not be processed together, but a separation of several hours affects reflectance in the image due to change in sun angle and even atmospheric conditions. Using a reference solar zenith angle partially rectifies these considerations.

**Figure 9:**
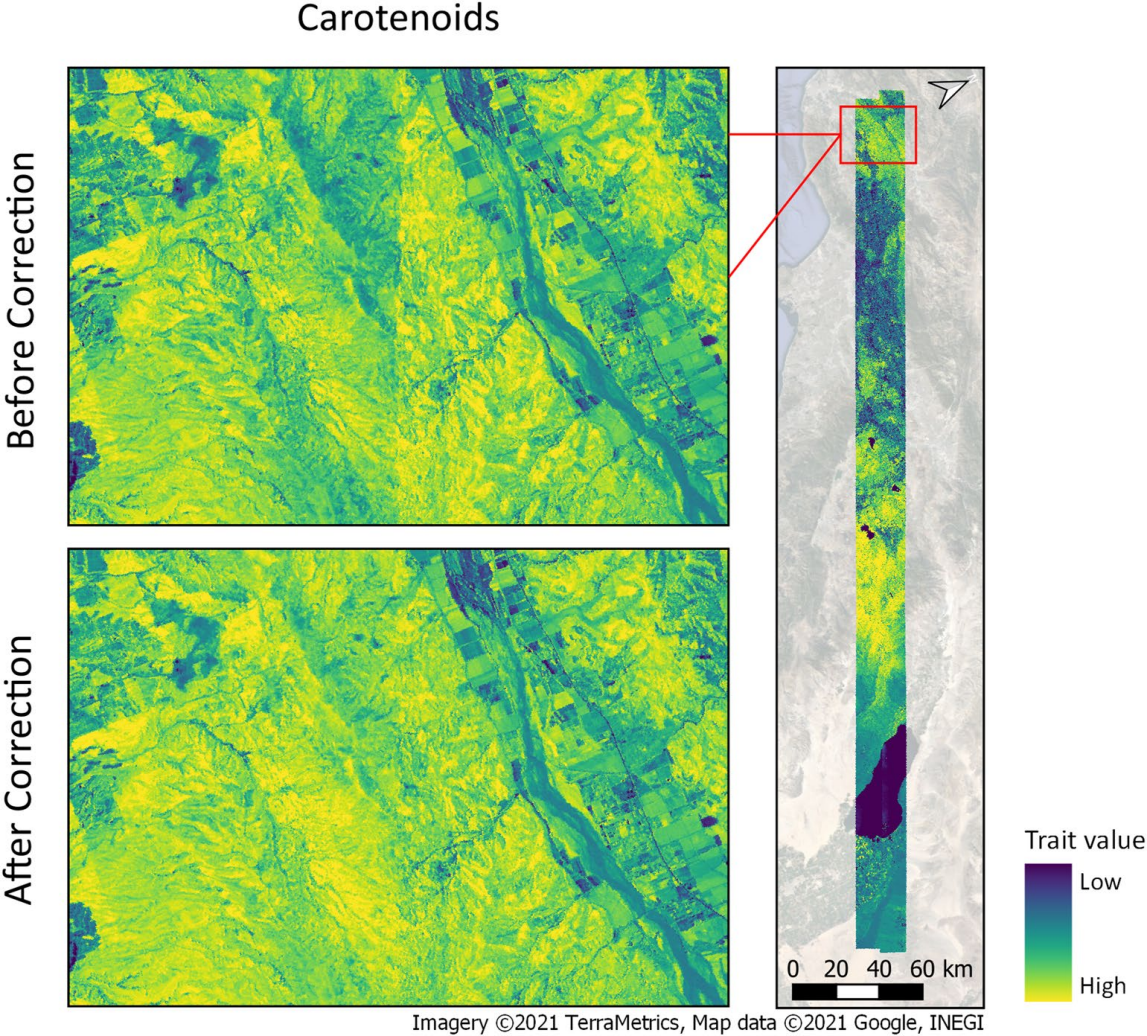
For illustration, a trait map of carotenoids is shown for uncorrected and bidirectional reflectance distribution function‐corrected data from two overlapping Southern California flightlines. A clear boundary line runs through the center of the map derived from uncorrected imagery.

Our overall objective for BRDF corrections is to reduce the influence of BRDF on derived maps, such as vegetation composition, fractional cover, disturbance, stress and functional traits. After an appropriate BRDF correction, algorithms that address ecological questions can be applied to generate maps that can be mosaicked with minimal seams. Such applications depend on consistent spectral reflectance across flightlines, and a method such as FlexBRDF can greatly reduce discontinuities between flightlines, as illustrated with a vegetation trait map derived for carotenoids following Singh et al. (2015); (Figure 9). Some methods are more sensitive to BRDF than others, for example, trait mapping (Singh et al., 2015) and quantification of biodiversity metrics (Féret & Asner, 2014), while methods that leverage unmixing or spectral angles are less sensitive to per pixel brightness effects.

Our code for FlexBRDF has been designed to be adaptable to a range of environments. However, it is currently optimized for vegetation and was tested on flight boxes that had reasonable vegetation cover. Although we demonstrate FlexBRDF using a parameterization that we found worked well across these environments, users of FlexBRDF can tune the approach to different kernel combinations, vegetation index, binning approach (the code also enables using a classification layer rather than NDVI bins), and reference solar zenith angle. Future work will include an optimization method for kernel selection, which may serve to expand the effectiveness of our approach to regions not focused on here, such as deserts and wetlands. FlexBRDF is part of a larger suite of open‐source Python tools for processing hyperspectral imagery available at https://github.com/EnSpec/hytools.

## 6 Conclusion

Current and forthcoming spaceborne imaging spectroscopy missions such as SBG will require extensive airborne data sets for calibration and validation. Data sets of derived products that exhibit artifacts of BRDF and other effects (e.g., Figure 9) may be problematic for integration with these new spaceborne data. FlexBRDF will allow users to mosaic maps of vegetation properties derived from imaging spectroscopy on groups of adjacent airborne flightlines without stark image boundaries created by BRDF effects. We demonstrate that FlexBRDF is highly applicable for airborne hyperspectral imagery covering diverse vegetated environments. However, our open source code is written such that users can implement and test a wide range of BRDF approaches. Our final recommendation is to group the images, stratify the data with 18 NDVI bins, use the solar zenith angle from the flight box center point as a reference, and smooth the coefficients with linear interpolation. While these parameters worked well on most sites (although Yosemite may benefit from a different solar zenith reference), the method is flexible and the user may adjust the parameters to fit their needs.

## Supporting information

Supporting Information S1

Table S1

Table S2

## Data Availability

The research presented here results from testing conducted over multiple years with support from multiple sources. AVIRIS‐Classic, AVIRIS‐NG, and NEON imagery were accessed from their respective data portals: https://aviris.jpl.nasa.gov/dataportal/, https://avirisng.jpl.nasa.gov/dataportal/, and (https://data.neonscience.org/).
