# Supplementary material for: FlexBRDF: A Flexible BRDF Correction for Grouped Processing of Airborne Imaging Spectroscopy Flightlines
Source: J Geophys Res Biogeosci. 2022 Jan 24;127(1):e2021JG006622. doi: 10.1029/2021JG006622 (PMC9286663; doi:10.1029/2021JG006622)
Supplement: Supplementary file 1 — Supporting Information S1 [file JGRG-127-0-s003.pdf]

JGR: Biogeosciences

Supporting Information for

FlexBRDF: A Flexible BRDF Correction for Grouped Processing of Airborne Imaging Spectroscopy Flightlines

Natalie Queally[1], Zhiwei Ye[1], Ting Zheng[1], Adam Chlus[1], Fabian Schneider[2], Ryan P. Pavlick[2], Philip A. Townsend[1]

[1]Department of Forest and Wildlife Ecology, University of Wisconsin-Madison, 1630 Linden Drive, Madison, WI 53706, USA. [2]Jet Propulsion Laboratory, California Institute of Technology, 4800 Oak Grove Drive, Pasadena, CA 91011, USA.

Contents of this file

Figures S1 to S11

Tables S2 and S4

Text S1

Additional Supporting Information (Files uploaded separately)

Captions for Tables S1 and S3

Introduction

The supporting tables and figures show further data exploration of BRDF-corrected imagery that is not crucial to the conclusions of the paper but provides further context for processing decisions. These include illustration of kernel, smoothing, and NDVI binning effects. These also include extra visuals for additional transects and secondary sites.

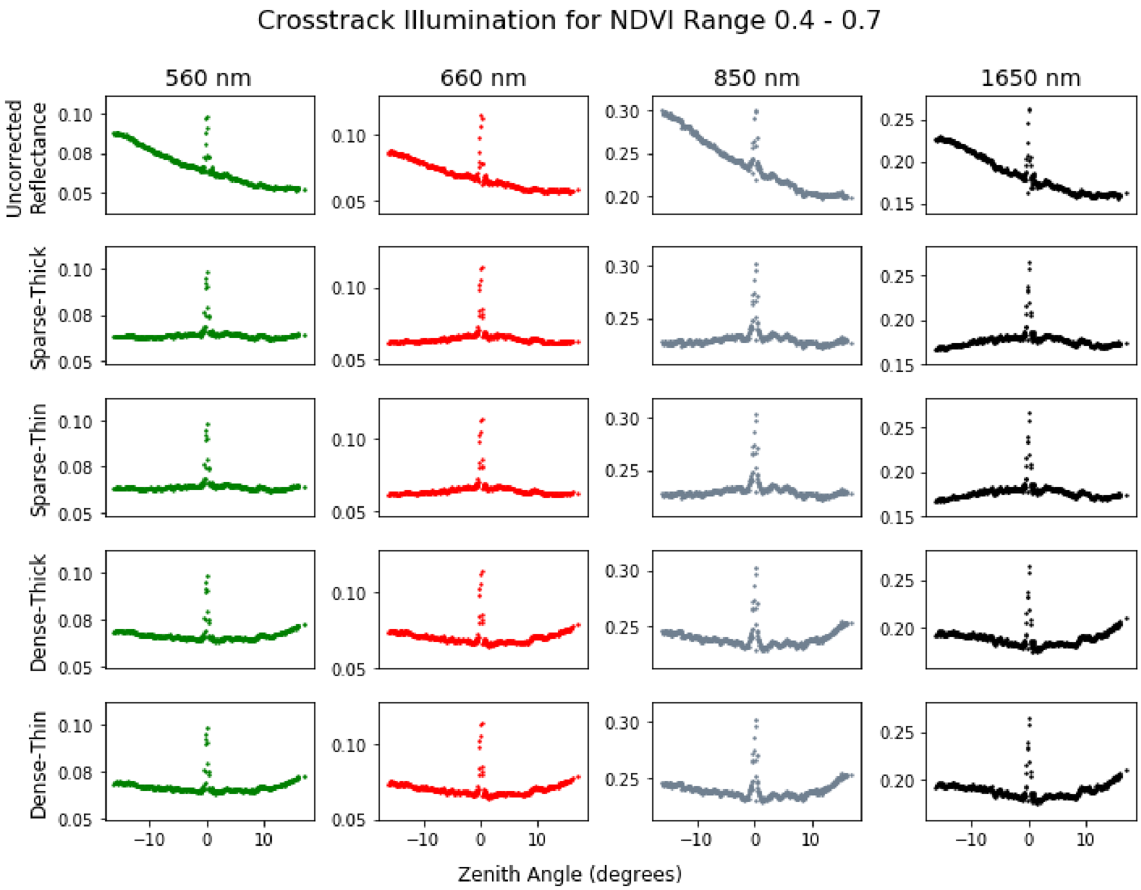

Figure S1. Reflectance values for four wavelengths within the 0.4 - 0.7 NDVI range for a flightline over Yosemite. The top row shows uncorrected reflectance plotted by sensor zenith angle, with a clear trend of decreasing reflectance from left to right across the image, the result of BRDF. Corrections using the dense kernel show an overcorrection, where pixels with sensor zenith angle > 0 trend upwards. Corrections using the sparse kernel show more level values across the image. The spike in reflectance at nadir (zenith angle 0) is an artifact of atmospheric correction.

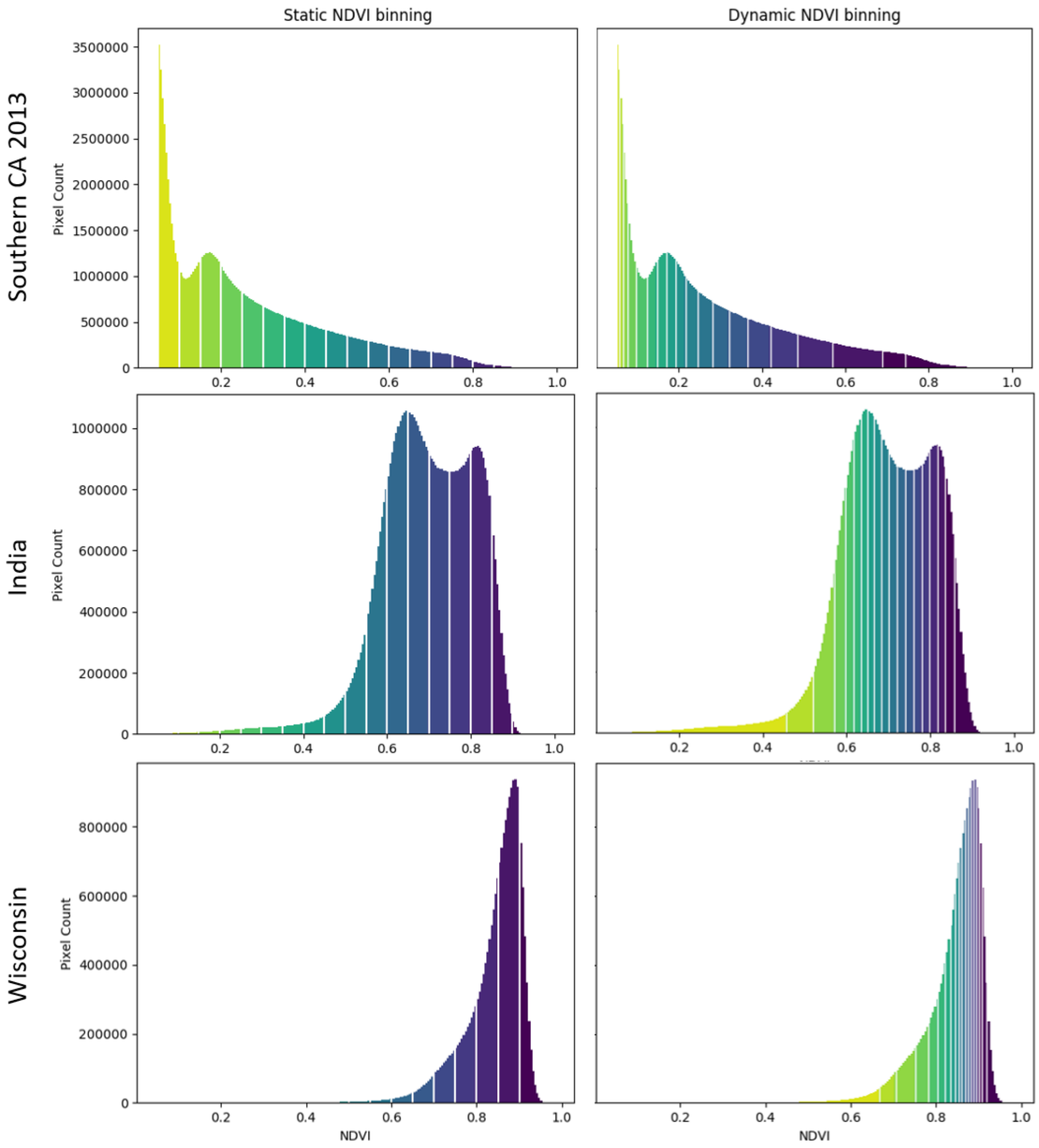

Figure S2. Dynamic bins were tested for images in the Wisconsin NEON box, where the NDVI range was largely restricted to upper values. The difference in NDVI divisions is shown for static and dynamic methods, and NDVI distribution is shown for Southern California 2013, Yosemite, and Wisconsin boxes.

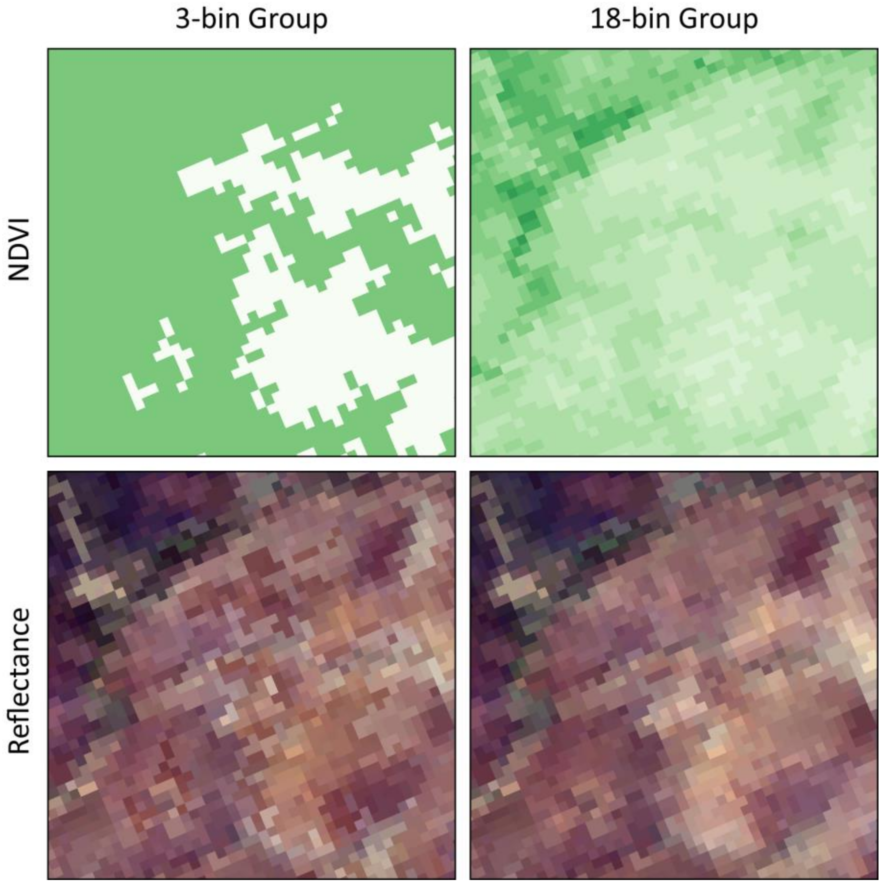

Figure S3. Sharper edges occur between NDVI bins in the 3-bin approach corrected image than the 18-bin approach. Darker green indicates higher NDVI, and the image stretch is identical for reflectance images.

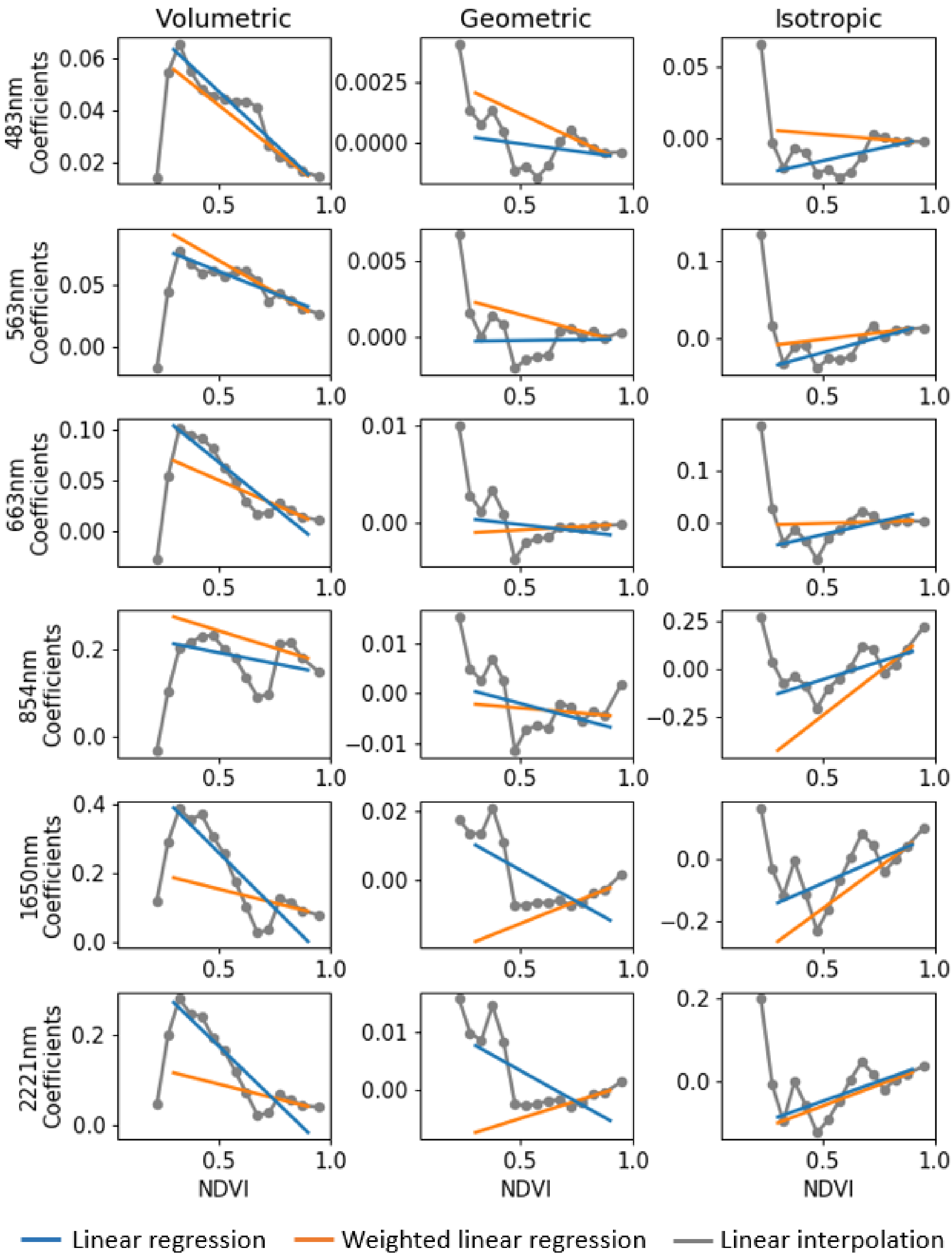

Figure S4. Tested smoothing methods for the BRDF coefficients included linear regression, weighted linear regression, and linear interpolation. These methods are visualized for six wavelengths for the Southern California 2013 flight box.

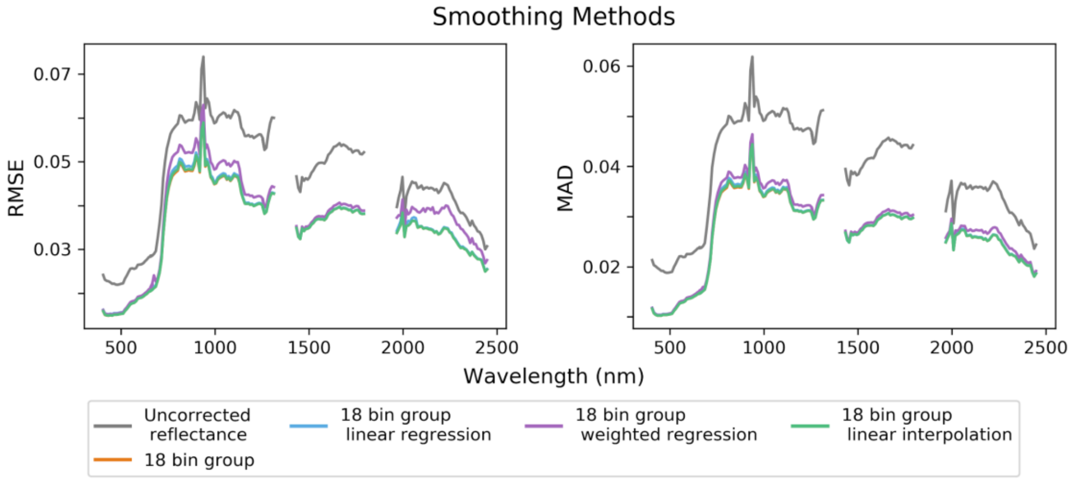

Figure S5. Smoothing methods showed little variation in overlap assessment metrics.

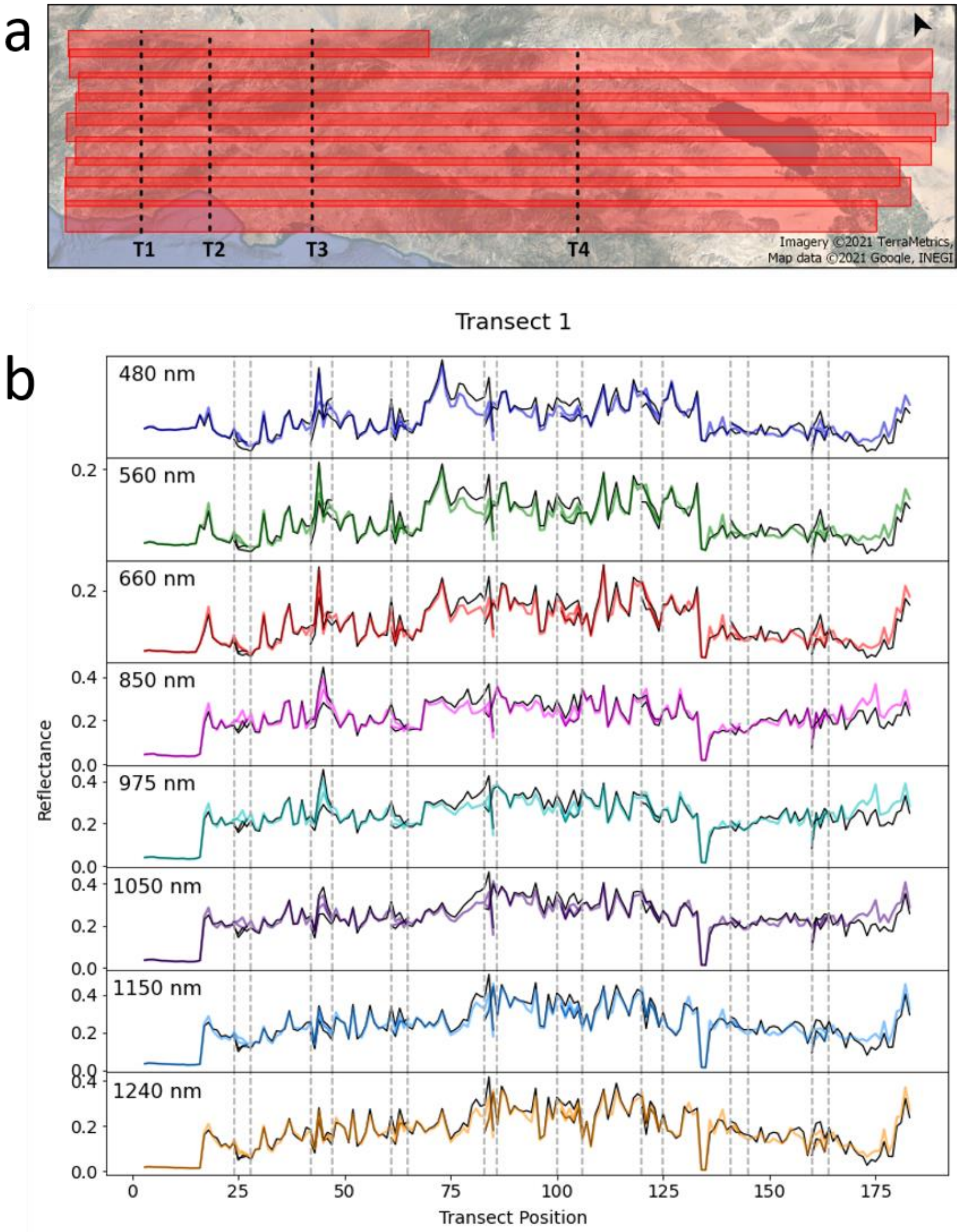
a
T1
T2
T3
T4
Imagery ©2021 TerraMetrics,
Map data ©2021 Google, INEGI
b
Transect 1
480 nm
560 nm
660 nm
850 nm
975 nm
1050 nm
1150 nm
1240 nm
Reflectance
Transect Position

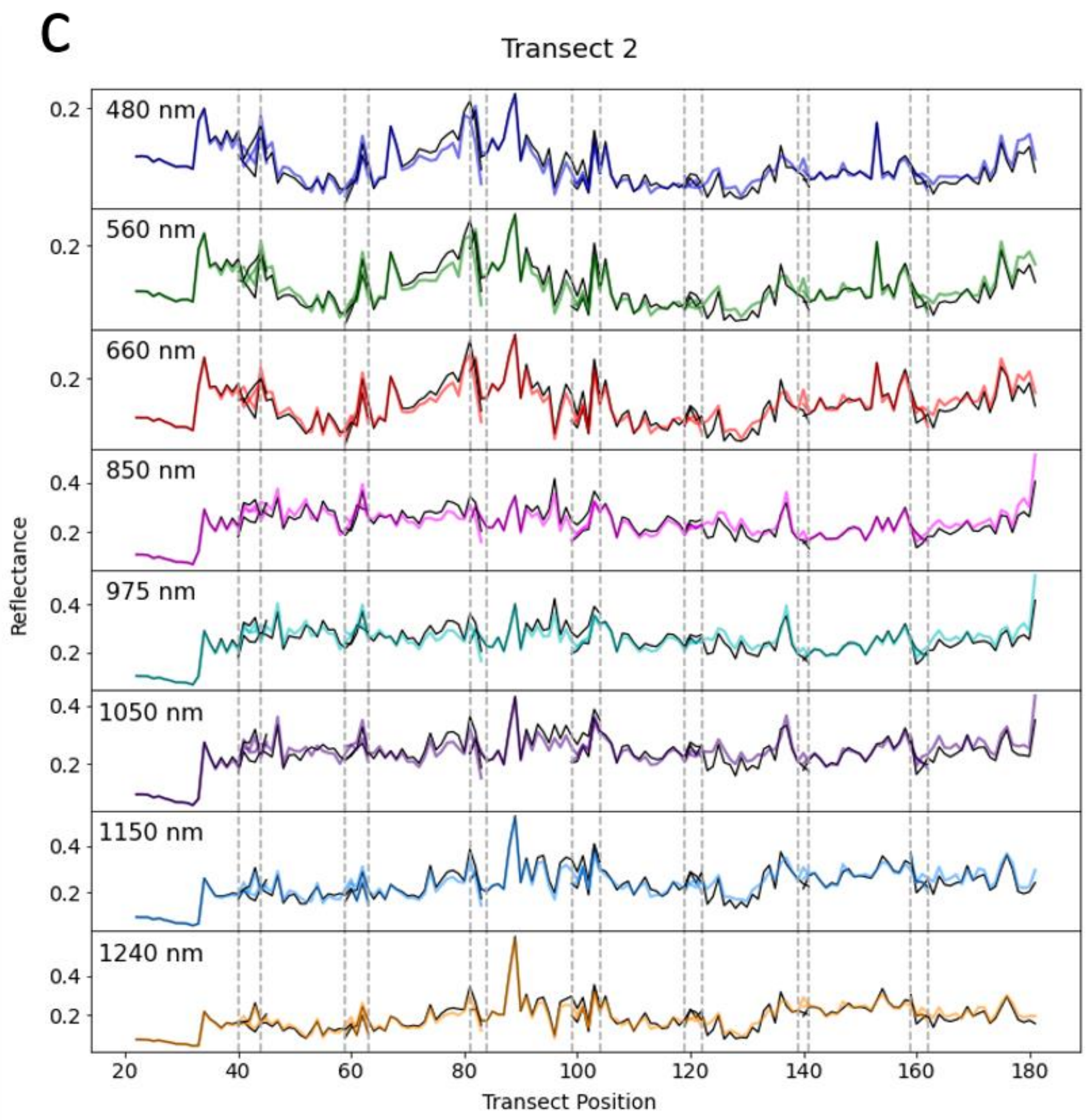
C
Transect 2
480 nm
560 nm
660 nm
850 nm
975 nm
1050 nm
1150 nm
1240 nm
Reflectance
Transect Position
0.2
0.4
20
40
60
80
100
120
140
160
180

d

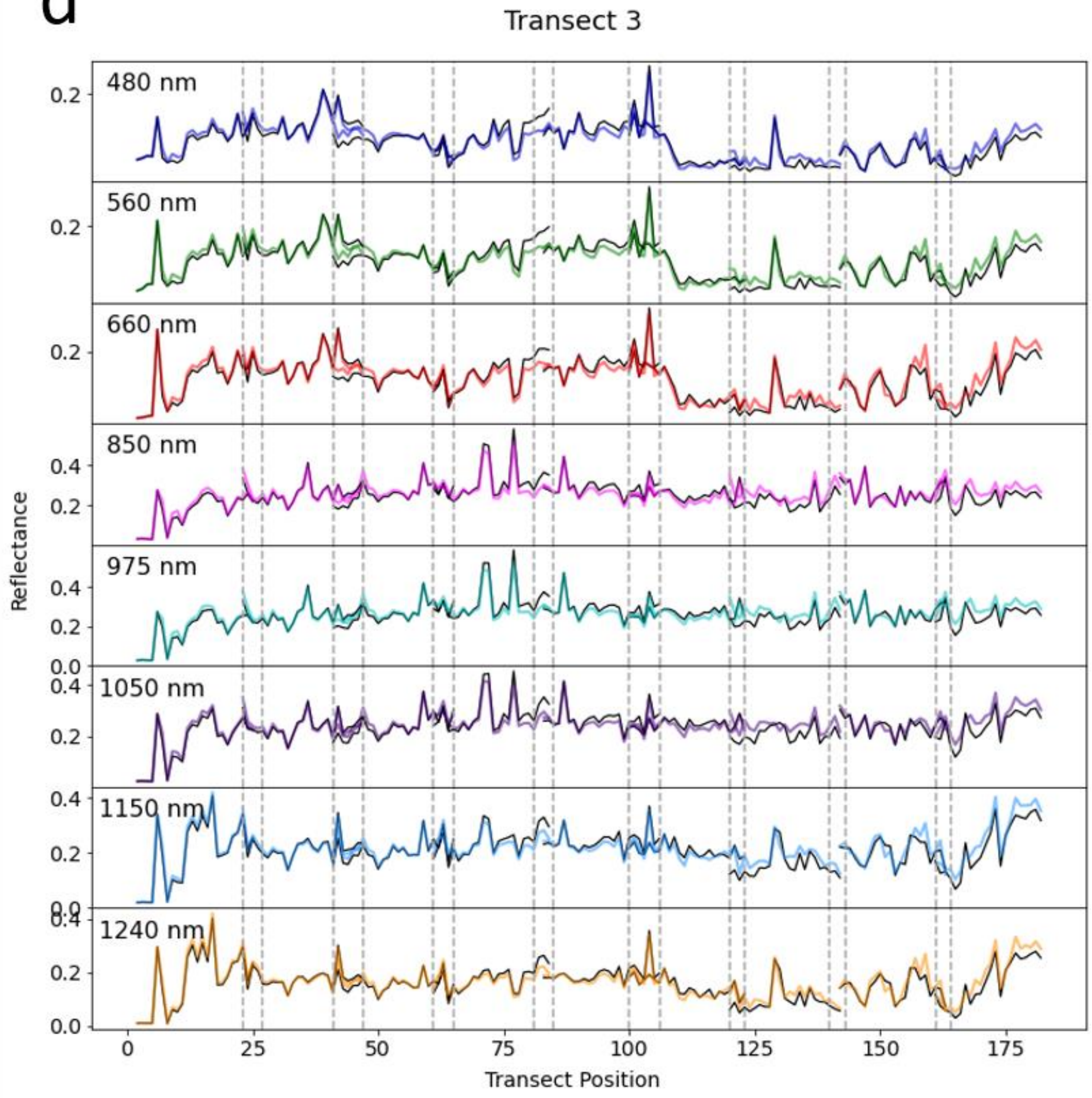

Transect 3
480 nm
560 nm
660 nm
850 nm
975 nm
1050 nm
1150 nm
1240 nm
Reflectance
Transect Position
0.2
0.4
0.0
0
25
50
75
100
125
150
175

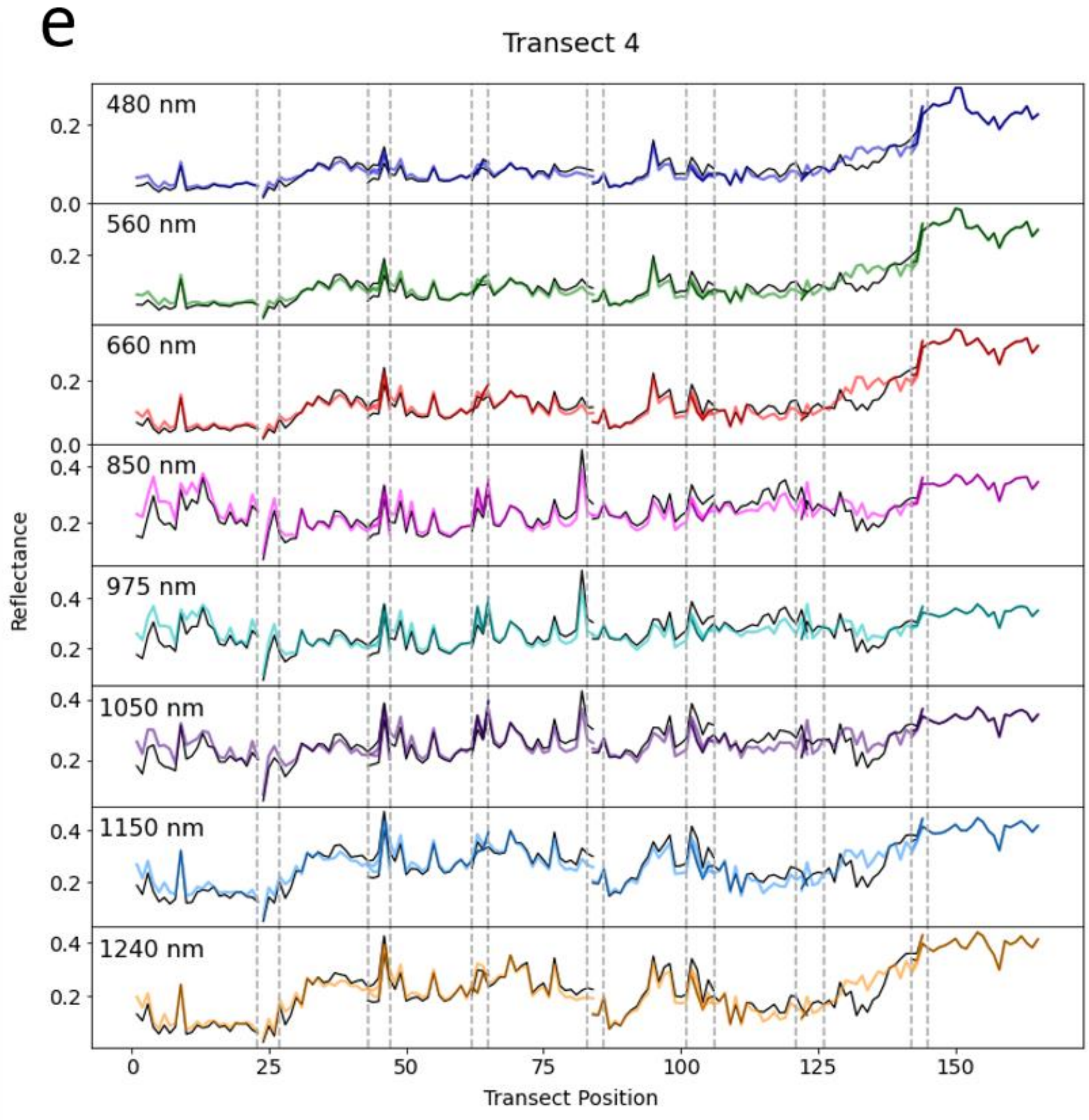

Figure S6. Four transects were drawn across the Southern California box (a). Average reflectance is shown across Transect 1 (b), with overlapping areas marked (dashed gray lines). Corrected imagery (color) is compared to uncorrected (black). Transect 1 and remaining transects (c-e) from corrected imagery are more continuous or similar to transects from uncorrected imagery in overlap areas.

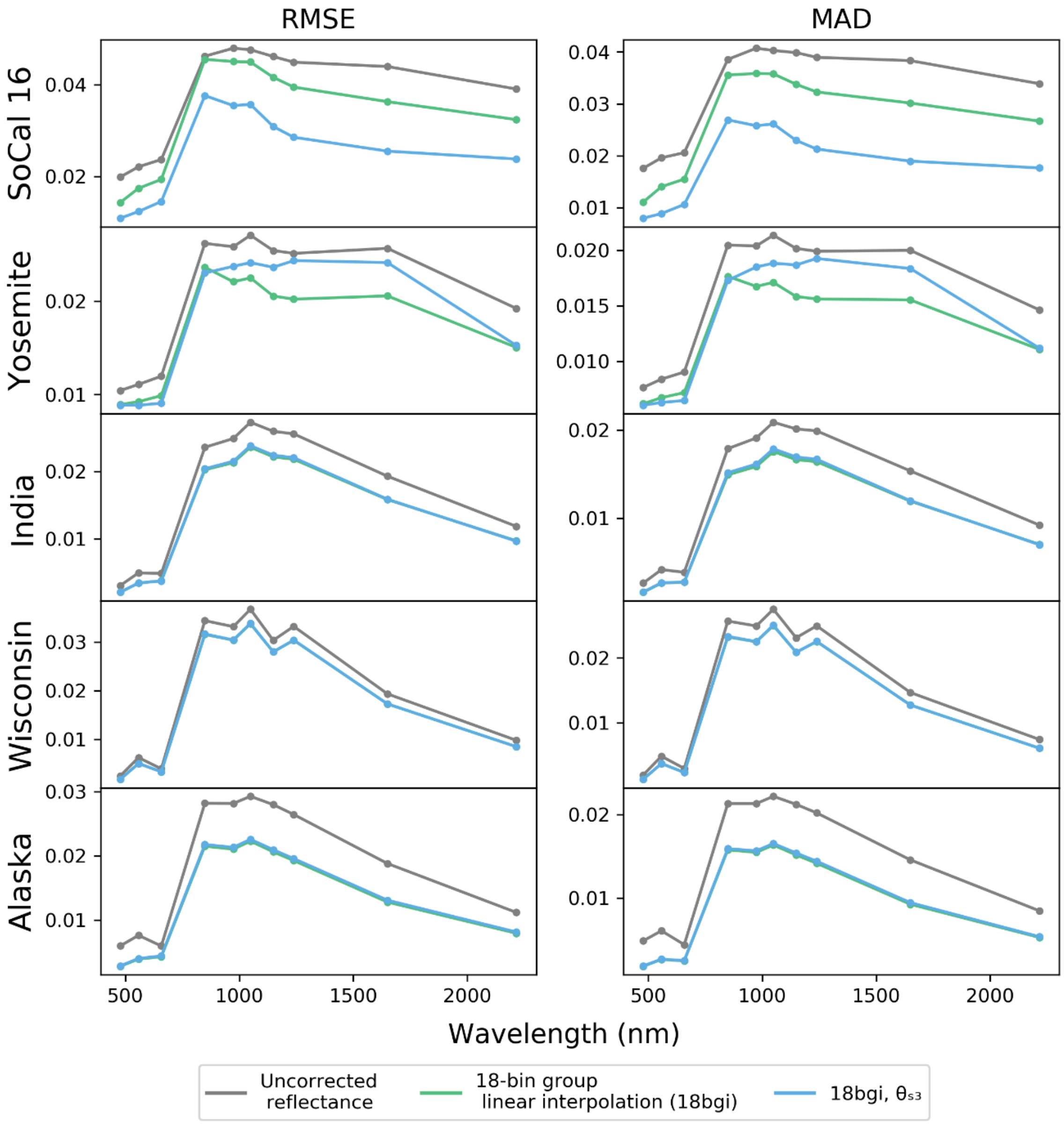

Figure S7. RMSE and MAD from overlapping areas across all sites shown for uncorrected reflectance and three BRDF correction methods. All corrections lowered RMSE and MAD, with little difference between methods.

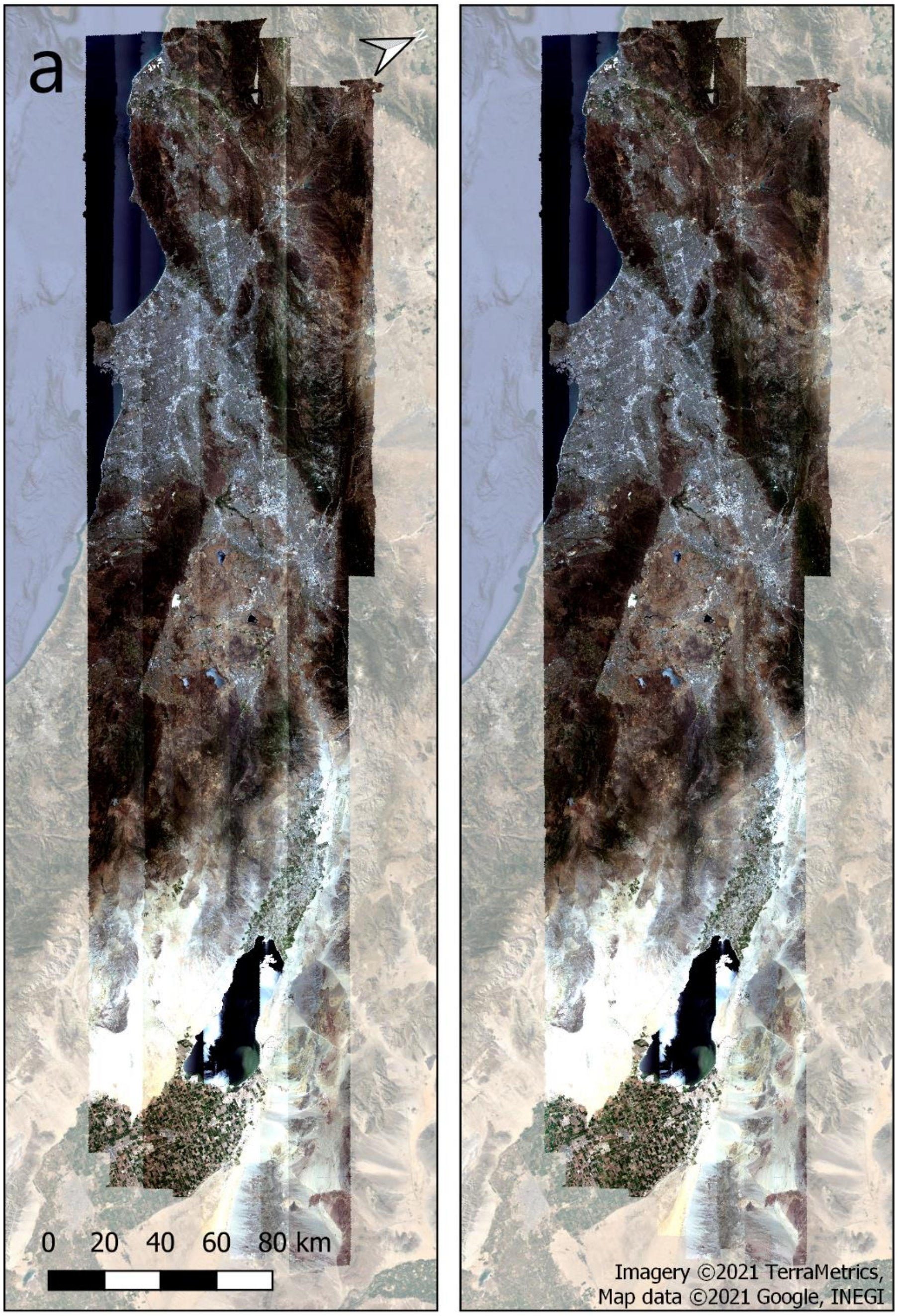
a
0 20 40 60 80 km
Imagery ©2021 TerraMetrics,
Map data ©2021 Google, INEGI

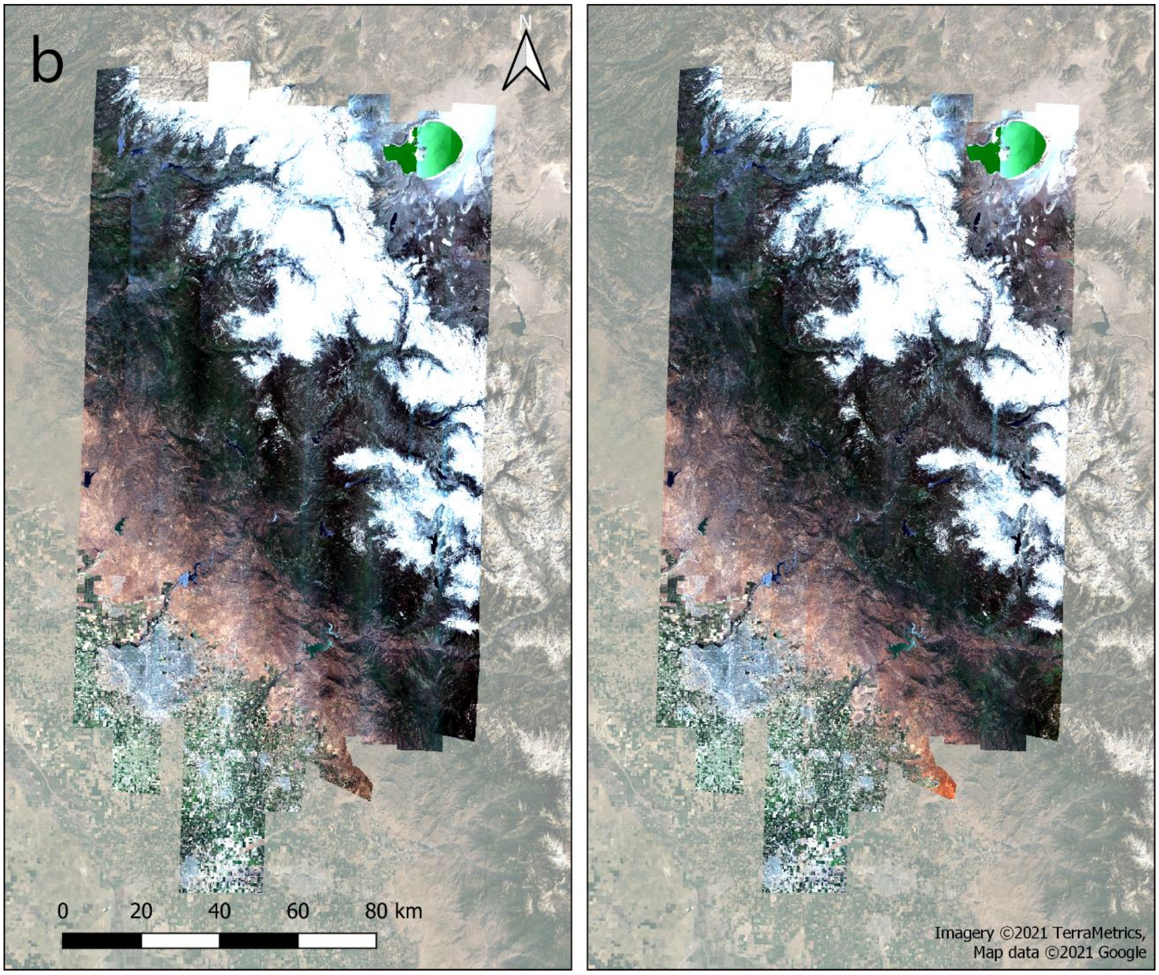
b
N
0
20
40
60
80 km
Imagery ©2021 TerraMetrics,
Map data ©2021 Google

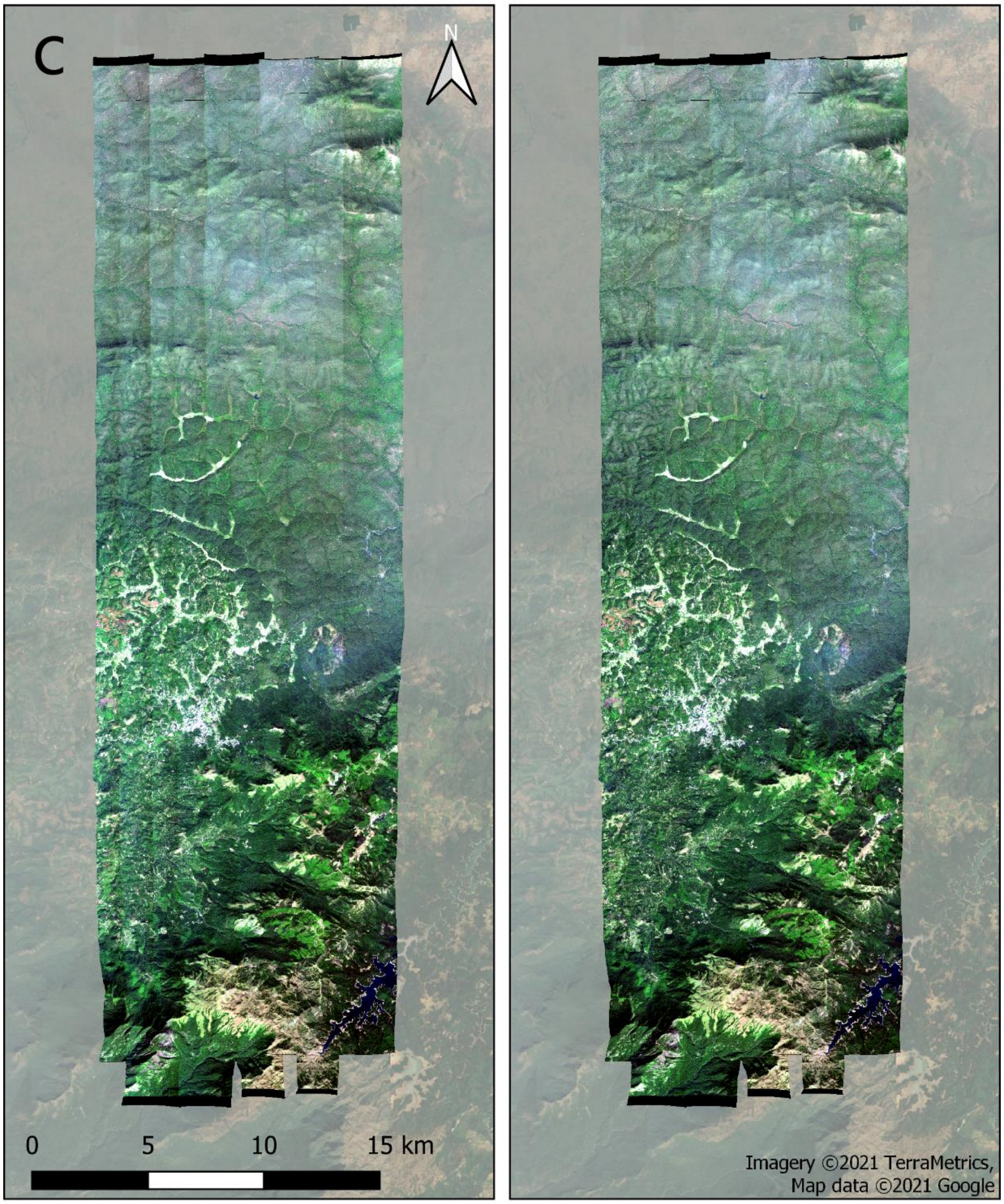
C
N
0
5
10
15 km
Imagery ©2021 TerraMetrics,
Map data ©2021 Google

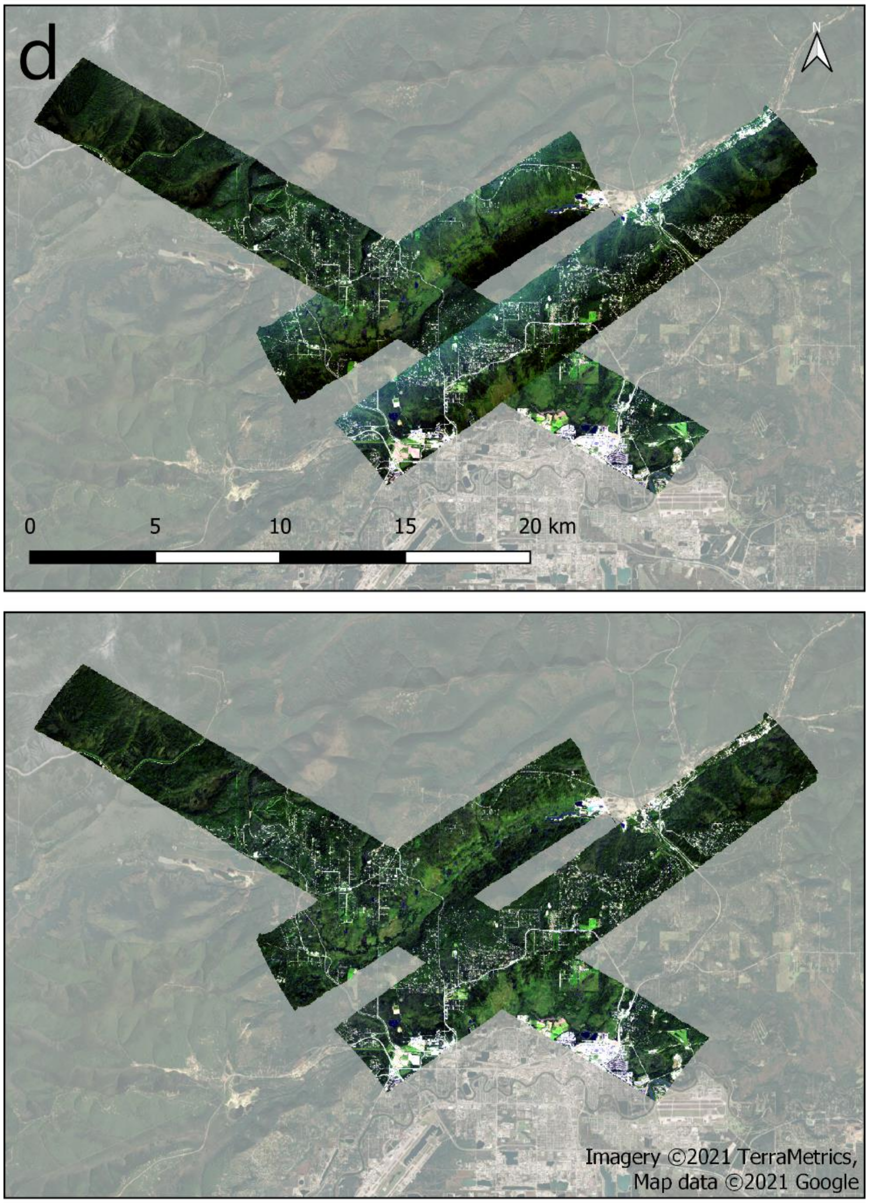

Figure S8. Before (left/top) and after (right/bottom) BRDF correction (18 bin group, interpolation, θ_{s3}) mosaics for Southern California 2016 (a), Yosemite (b), India (c), and Alaska (d).

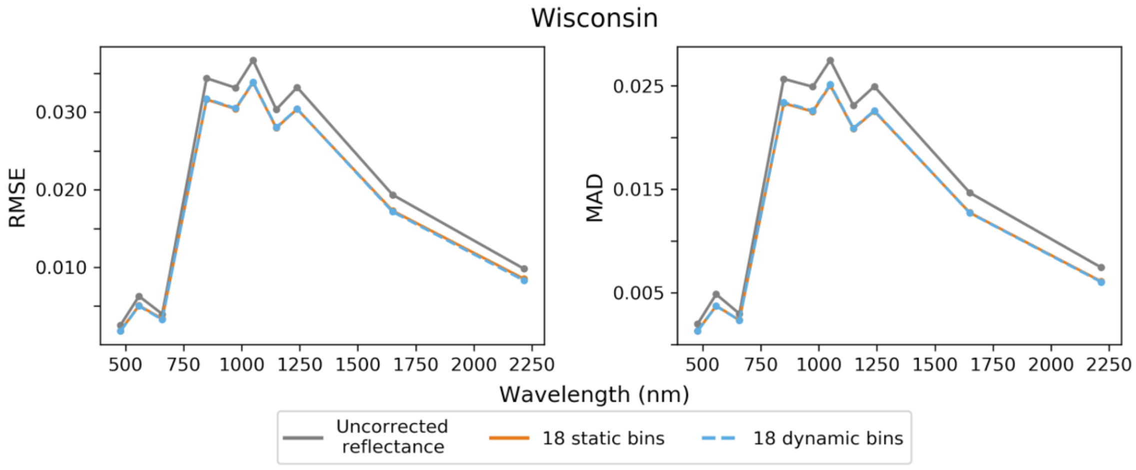

Figure S9. RMSE and MAD derived from static vs. dynamic binning showed no significant difference for the NEON Wisconsin box.

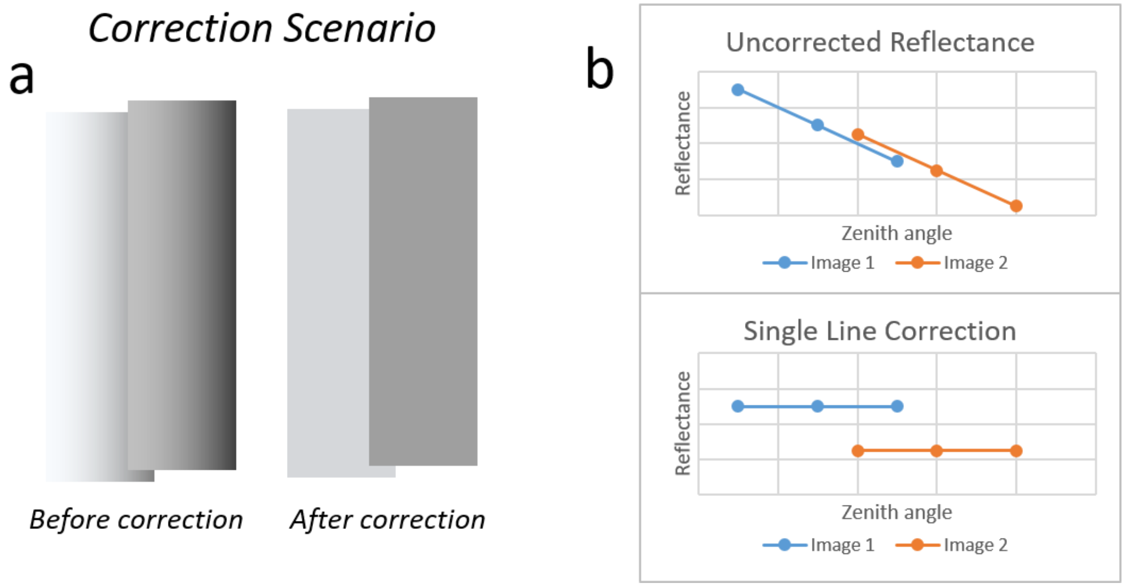

Figure S10. Due to the gap in acquisition time in adjacent images that we see in the Southern California box, it is possible that two overlapping areas could be closer in reflectance before correction (as compared to single line correction) due to BRDF effects. In the above scenario (a), both images have the same direction of brightness gradient (lighter to darker from left to right) as a result of BRDF effects. However, different illumination conditions due to differing acquisition times has created a relative difference in overall brightness across the gradient for each image. Plots (b) show BRDF effects on uncorrected reflectance (variation in brightness across track), and corrected reflectance after single line BRDF correction (constant brightness across track). In this scenario, the overlapping areas are more similar in the uncorrected imagery due to the BRDF effects. The group correction precludes this issue by drawing a random sample across the entire box, which accounts for the varying illumination conditions experienced by the flightlines across several hours of data acquisition.

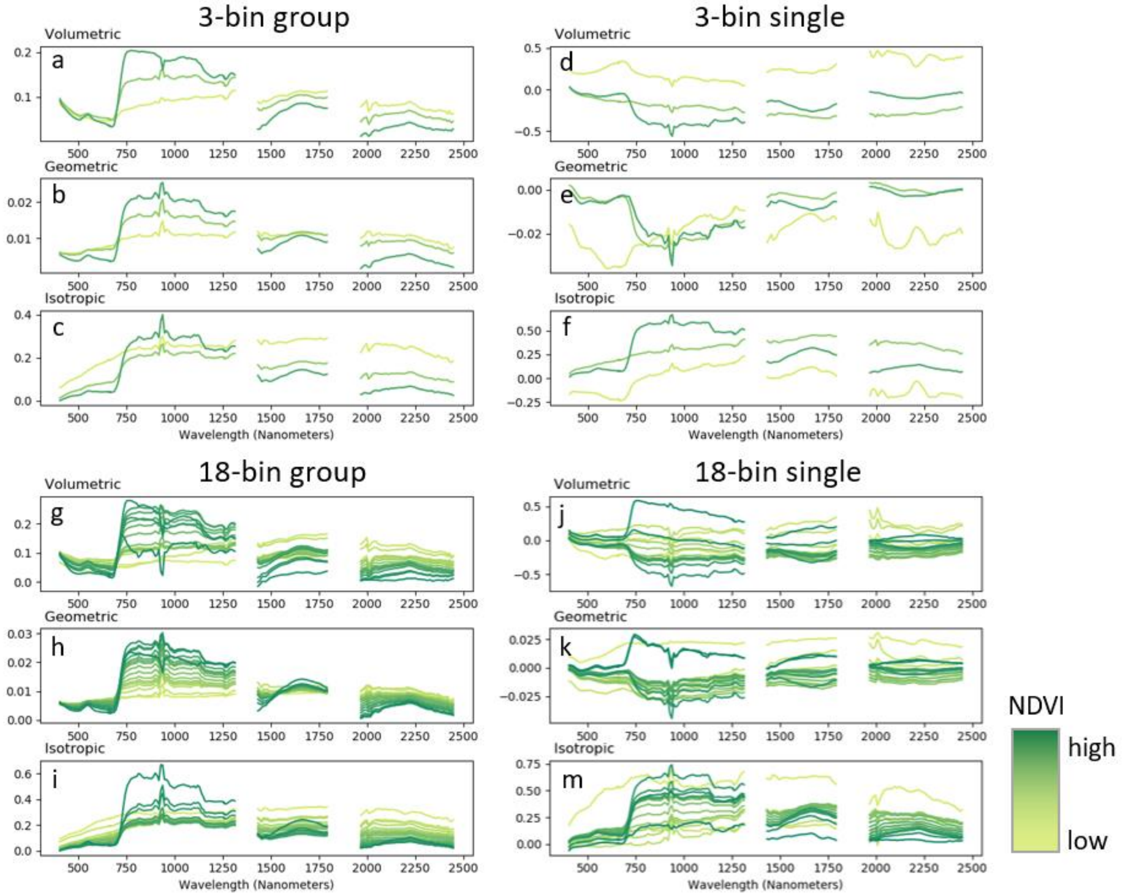

Figure S11. Geometric, volumetric, and isotropic coefficient values shown for single and grouping methods. BRDF coefficients for grouping methods more closely resemble a vegetation spectrum as NDVI increases.

Table S1. (Separate file) List of flightlines used from each flight box.

Table S2. NDVI value divisions for the static NDVI bin approach.

# bins	Bin boundaries
3	0.3, 0.7
8	0.2, 0.3, 0.4, 0.5, 0.6, 0.7, 0.8
18	0.1, 0.15, 0.2, 0.25, 0.3, 0.35, 0.4, 0.45, 0.5, 0.55, 0.6, 0.65, 0.7, 0.75, 0.8, 0.85, 0.9

Table S3. (Separate file) Record of change in RMSE and MAD averaged across all overlap areas in a flight box for 10 bands. Images were corrected with 18-bin group, interpolation, θ_{s3} approach. Negative values indicate lower RMSE/MAD in the corrected imagery compared to the uncorrected.

Table S4. Comparison of different BRDF methods for correction of imaging spectroscopy data.

Source	Method Name	Kernel Selection	Topo Corr. (Y/N)	Grouping method	Pre-classification	Reference SZA
Queally et al., 2021 (this paper)	FlexBRDF	Li-Sparse, Ross-Thick	Y	Stratified random sampling across all lines	Continuous index (NDVI bins)	Flight box average
Jia et al., 2020	RT-BRDF	Li-Transit-Reciprocal, Ross-Thick-Maignan	Y	NA	Support vector machine: coniferous forest, broadleaf forest, bare soil, urban, unclassified	Calculated by date, average longitude and latitude, and median flightline acquisition time
Jensen et al., 2018	ARGC	NA	N	NA	Band, NDVI, and NDWI thresholding: water 1, water 2, water 3, vegetation, impervious/cloud/shadow	NA
Schläpfer et al., 2015	BREFCOR	Li-Sparse-Reciprocal, Ross-Thick	Y	Correction models averaged across lines	Continuous BRDF cover index (BCI)	NA
Weyermann et al., 2015	NA	Ross-Li Kernel combination determined dynamically	Y	NA	Relative abundance of forest, low vegetation, and soil derived from spectral unmixing	Flight box average
Colgan et al., 2012	NA	Li-Dense, Ross-Thick	N	Stratified random sampling across box mosaic	NDVI and NIR filters: well-lit vegetation, shaded veg, grass, soil, other	Flight box average
Collings et al., 2010	NA (suite of correction models)	Li-Sparse, Ross-Thick	N	NA	NA	NA

Text S1. Description of spectral matching technique.

We report statistics for pixels in overlapping areas of adjacent flightlines as metrics of BRDF model performance. Specifically, a BRDF correction should reduce the differences in reflectance between pixels in overlapping areas. Spatial misalignment between overlap areas was addressed using spectral matching. For each pair of overlapping images, we identified a base and a target image. We delineated contiguous 12 x 12 pixel areas in the overlapping region in the base image, and used a moving window on the target image at a maximum search distance of 15 pixels in the along- and across-track directions to identify the corresponding areas that maximized the correlation of reflectance between the base and target windows.
