# Supplementary material for: FlexBRDF: A Flexible BRDF Correction for Grouped Processing of Airborne Imaging Spectroscopy Flightlines
Source: J Geophys Res Biogeosci. 2022 Jan 24;127(1):e2021JG006622. doi: 10.1029/2021JG006622 (PMC9286663; doi:10.1029/2021JG006622)
Supplement: Supplementary file 2 — Table S1 [file JGRG-127-0-s001.docx]

# Supporting Information

### **Table S1.**

| Flight box | Flightlines |
| --- | --- |
| Southern California 2013 | f130522t01p00r05  f130522t01p00r06  f130522t01p00r07  f130522t01p00r08  f130522t01p00r09  f130522t01p00r10  f130522t01p00r11  f130522t01p00r12  f130522t01p00r13 |
| Southern California 2016 | f160616t01p00r08  f160616t01p00r09  f160616t01p00r10  f160616t01p00r11  f160616t01p00r12  f160616t01p00r13  f160616t01p00r14  f160616t01p00r15  f160616t01p00r16  f160616t01p00r17 |
| Yosemite | f170607t01p00r06  f170607t01p00r07  f170607t01p00r08  f170607t01p00r09  f170607t01p00r10  f170607t01p00r11  f170607t01p00r12  f170607t01p00r13  f170607t01p00r14  f170607t01p00r15  f170607t01p00r16 |
| Fairbanks, AK | ang20180723t193953  ang20180723t194529  ang20180723t195338 |
| Mudumalai, India | ang20160105t051247  ang20160105t052624  ang20160105t053937  ang20160105t055235  ang20160105t060629  ang20160105t062045  ang20160105t063459  ang20160105t064945  ang20160105t070345  ang20160105t073139 |
| Chequamegon,  WI | NEON_D05_CHEQ_DP1_20170911_181003  NEON_D05_CHEQ_DP1_20170911_181559  NEON_D05_CHEQ_DP1_20170911_182212  NEON_D05_CHEQ_DP1_20170911_182822  NEON_D05_CHEQ_DP1_20170911_183324 |
