# Supplementary material for: FlexBRDF: A Flexible BRDF Correction for Grouped Processing of Airborne Imaging Spectroscopy Flightlines
Source: J Geophys Res Biogeosci. 2022 Jan 24;127(1):e2021JG006622. doi: 10.1029/2021JG006622 (PMC9286663; doi:10.1029/2021JG006622)
Supplement: Supplementary file 3 — Table S2 [file JGRG-127-0-s002.docx]

### **Supporting Information**

### **Table S2.**

| Site | Wavelength (nm) | RMSE change | MAD change |
| --- | --- | --- | --- |
| Southern California 2013 | 478 | -0.009 | -0.011 |
|  | 558 | -0.011 | -0.013 |
|  | 658 | -0.012 | -0.013 |
|  | 849 | -0.014 | -0.017 |
|  | 974 | -0.018 | -0.021 |
|  | 1049 | -0.018 | -0.021 |
|  | 1149 | -0.02 | -0.023 |
|  | 1239 | -0.021 | -0.023 |
|  | 1650 | -0.021 | -0.022 |
|  | 2216 | -0.016 | -0.017 |
| Southern California 2016 | 478 | -0.009 | -0.01 |
|  | 558 | -0.01 | -0.011 |
|  | 658 | -0.009 | -0.01 |
|  | 849 | -0.009 | -0.012 |
|  | 974 | -0.013 | -0.015 |
|  | 1049 | -0.012 | -0.014 |
|  | 1149 | -0.015 | -0.017 |
|  | 1239 | -0.016 | -0.018 |
|  | 1650 | -0.018 | -0.019 |
|  | 2216 | -0.015 | -0.016 |
| Yosemite | 478 | -0.002 | -0.002 |
|  | 558 | -0.002 | -0.002 |
|  | 658 | -0.003 | -0.003 |
|  | 849 | -0.003 | -0.003 |
|  | 974 | -0.002 | -0.002 |
|  | 1049 | -0.003 | -0.003 |
|  | 1149 | -0.002 | -0.001 |
|  | 1239 | -0.001 | -0.001 |
|  | 1650 | -0.002 | -0.002 |
|  | 2216 | -0.004 | -0.003 |
| India | 478 | -0.001 | -0.001 |
|  | 558 | -0.001 | -0.001 |
|  | 658 | -0.001 | -0.001 |
|  | 849 | -0.003 | -0.003 |
|  | 974 | -0.003 | -0.003 |
|  | 1049 | -0.003 | -0.003 |
|  | 1149 | -0.004 | -0.003 |
|  | 1239 | -0.004 | -0.003 |
|  | 1650 | -0.003 | -0.003 |
|  | 2216 | -0.002 | -0.002 |
| Wisconsin | 478 | -0.001 | -0.001 |
|  | 558 | -0.001 | -0.001 |
|  | 658 | -0.001 | -0.001 |
|  | 849 | -0.003 | -0.002 |
|  | 974 | -0.003 | -0.002 |
|  | 1049 | -0.003 | -0.002 |
|  | 1149 | -0.002 | -0.002 |
|  | 1239 | -0.003 | -0.002 |
|  | 1650 | -0.002 | -0.002 |
|  | 2216 | -0.001 | -0.001 |
| Alaska | 478 | -0.003 | -0.003 |
|  | 558 | -0.004 | -0.003 |
|  | 658 | -0.002 | -0.002 |
|  | 849 | -0.006 | -0.005 |
|  | 974 | -0.007 | -0.006 |
|  | 1049 | -0.007 | -0.006 |
|  | 1149 | -0.007 | -0.006 |
|  | 1239 | -0.007 | -0.006 |
|  | 1650 | -0.006 | -0.005 |
|  | 2216 | -0.003 | -0.003 |
